# Oral Health-Related Quality of Life in Pediatric Subjects with Molar Incisor Hypomineralization: An Umbrella Review of Domain-Specific Impact and Treatment-Related Changes

**DOI:** 10.3390/children13091235

**Published:** 2026-09-12

**Authors:** Massimo Pisano, Giuseppe Sangiovanni, Eugenio Frucci, Michela Scorziello, María del Carmen Villanueva-Vilchis, Giuseppina De Benedetto

**Affiliations:** 1 Department of Medicine, Surgery and Dentistry, “Scuola Medica Salernitana”, University of Salerno, Baronissi, 84081 Salerno, Italy; gsangiovanni7@gmail.com (G.S.); eugenio.frucci@gmail.com (E.F.); scorziellomichela96@gmail.com (M.S.); 2 Department of Oral Public Health, National School of Higher Studies (ENES)-Leon, National Autonomous University of Mexico, Leon 37684, Guanajuato, Mexico; cvillanueva@enes.unam.mx

**Keywords:** MIH, molar incisor hypomineralization, OHRQoL, oral health-related quality of life, quality of life, children, pedodontics, pediatric dentistry

## Abstract

**Highlights:**

**What are the main findings?**

- The present umbrella review synthesized six systematic reviews, showing an overall child self-reported OHRQoL percentage impact index of 21.76% in pediatric subjects with MIH, with the highest domain-specific impact across both child and parents/caregivers observed in the oral domain (26.43%), followed by the psychological (20.25%), functional (19.50%), and social (11.75%) domains.
- Children consistently reported a greater overall perceived impact on OHRQoL than their parents or caregivers (21.76% vs. 10.33%), revealing marked parental underestimation; furthermore, early mixed dentition showed a higher perceived impact than late mixed/permanent dentition (26.77% vs. 14.43%), while exploratory findings on a limited subsample (*n* = 494) suggested a potential deterioration in percentage scores considering MIH severity; no systematic review assessed lesion extent or distribution patterns.

**What is the implication of the main finding?**

- The notable discrepancy between child- and parent/caregiver-reported OHRQoL outcomes underlines the need for dentists and pediatric dentists to account for self-reported feedback from children, while also enhancing parents’ oral health literacy and fostering knowledge regarding the broader psychosocial and social implications of MIH.
- Targeted dental interventions (including preventive, esthetic, and restorative) may effectively improve overall and domain-specific OHRQoL, not only for oral function and symptom management, but also for pediatric emotional and social well-being; however, as evidence stems from a single systematic review (*n* = 491) with heterogeneous follow-ups, definitive conclusions regarding long-term OHRQoL treatment impact cannot be drawn.

**Abstract:**

Background/Objectives: Molar incisor hypomineralization (MIH) is a qualitative enamel defect affecting pediatric populations, predisposing to hypersensitivity, caries, and post-eruptive breakdown, and potentially impacting oral health-related quality of life (OHRQoL). Therefore, the primary aim of the present umbrella review was to evaluate the overall OHRQoL among pediatric subjects with MIH, as well as individual domains from both children and parents. Secondary aims were to evaluate the influence of MIH severity, extent/distribution pattern, age, and dentition status on overall- and domain-specific OHRQoL outcomes, and to assess the impact of different dental treatments on OHRQoL. Methods: Following PRISMA guidelines and PROSPERO registration, six systematic reviews were included. To address metric heterogeneity, mean scores were normalized into a 0–100% descriptive percentage impact index. Treatment-related changes were calculated as percentage improvements. Results: A total of 4028 respondents (3193 children and 835 parents/caregivers) were evaluated for OHRQoL. The overall impact on children and parents was highest in the oral domain (26.43%), followed by the psychological (20.25%), functional (19.50%), and social (11.75%) domains. Parental underestimation of MIH was consistently observed across all domains. Children in early mixed dentition reported a higher perceived impact than those in late mixed/permanent dentition (26.77% vs. 14.43%). In an exploratory subsample (*n* = 494), MIH severity appeared to impact OHRQoL, particularly in oral and functional domains. Overall, post-treatment OHRQoL improved across all domains, although evaluated in a limited sample (*n* = 491). Conclusions: MIH showed a relevant impact on pediatric OHRQoL. Notably, children consistently report a greater perceived impact than parents, suggesting potential parental underestimation. However, findings regarding dentition stage, MIH severity, and post-treatment improvements should be interpreted with caution, as heterogeneity in assessment tools, limited subsamples, and variable follow-up intervals precludes definitive conclusions.

## 1. Introduction

Molar incisor hypomineralization (MIH) is a developmental enamel defect affecting the pediatric population, involving one or more permanent first molars and often the permanent incisors [1,2,3]. Recent evidence suggests that clinically similar altered features may also affect other permanent teeth, including canines, premolars, and second molars, potentially indicating a broader clinical phenotype of the condition [3,4].

The global prevalence of MIH is estimated at 13–14%, although wide variations have been reported across populations and geographic regions [5,6]. These differences may be attributed, at least in part, to the use of different diagnostic criteria and classification systems, which may contribute to an underestimation of the condition’s actual prevalence. Despite growing scientific interest, the etiopathogenesis of MIH has not yet been fully elucidated, and the currently available evidence supports a multifactorial origin arising from the interaction between genetic predisposition and environmental exposures that interfere with amelogenesis during enamel development [5,6,7,8].

Among the main proposed predisposing factors are prenatal factors, such as maternal complications and conditions arising during pregnancy; perinatal factors, including prematurity and low birth weight; and postnatal factors, such as systemic diseases in early childhood, recurrent febrile episodes, respiratory infections, exposure to antibiotics, and other conditions that could potentially alter the proper development of tooth enamel [6,7,9].

Enamel affected by MIH exhibits reduced mineral content, an increased protein matrix, and heightened porosity relative to healthy enamel [10,11,12]. These alterations impair the tissue’s mechanical integrity and diminish its functional properties [10,11,12]. Clinically, MIH presents as well-defined opacities of various sizes and colors, ranging from cream-white to yellow-brown, serving as the initial clinical indicator of the condition [1,6,7,13].

Teeth affected by MIH may later develop hypersensitivity, ranging from mild responses to thermal and tactile stimuli to spontaneous, persistent symptoms that may be severe enough to hinder oral hygiene, mastication, and routine dental interventions [14,15,16,17,18,19]. In more severe manifestations, enamel fragility can lead to post-eruptive breakdown (PEB), exposing the underlying dentin, causing coronal destruction, and resulting in progressive loss of dental tissue, potentially heightening susceptibility to caries [9,14,15,16]. The high organic matrix content and compromised quality of residual enamel may undermine the adhesion of restorative materials, complicating clinical management and elevating the risk of atypical, recurrent, or failed restorations, which may lead to tooth extraction [9,14,15,16].

In this context, oral health-related quality of life (OHRQoL) is an increasingly assessed outcome in dental research as it evaluates how oral health conditions affect an individual’s physical, functional, psychological, and social well-being, potentially enabling a more comprehensive assessment of the disease burden and its impact on daily life [14,20,21,22].

In pediatric patients with MIH, this approach may be particularly relevant, as the perceived oral condition can interfere with eating, speech, participation in school and recreational activities, peer relationships, and emotional development. Given the chronic nature of the condition and the potential frequent need for repeated treatments over time, assessing OHRQoL may represent an essential complement to traditional clinical outcomes.

However, the impact of MIH on OHRQoL may not be uniform across the affected pediatric individuals. It has been hypothesized that the severity of the lesions, the number of teeth involved, the involvement of the anterior region, the presence of hypersensitivity, and post-eruptive breakdown may influence perceptions of the disease in different ways [22,23,24,25]. Similarly, the patient’s age and stage of dentition may be significant determinants of quality of life. In younger children, indeed, the incomplete eruption of the first permanent molars may limit the full clinical manifestation of the condition, whereas in later stages of mixed and permanent dentition, the progression of lesions, the onset of complications, and the accumulation of dental experiences may result in a greater impact on quality of life. Similarly, involvement of the permanent incisors may amplify the psychological and social burden of the disease due to the esthetic implications associated with this location.

The assessment of OHRQoL in children involves the collection of information both directly from the child and through parents or caregivers [22,23,24,25]. Although both perspectives are considered complementary, it has been suggested that parents’ perceptions may not fully reflect the child’s subjective experience, leading to underestimation or, in some cases, overestimation of the condition’s impact [6,22].

Despite growing interest in the relationship between MIH and OHRQoL, the available evidence remains heterogeneous and, in part, conflicting [22,23,25,26,27,28,29]. Some studies have shown a relevant association between MIH and a decline in oral health-related quality of life, while others have found no clinically relevant differences compared to unaffected individuals [22,23,25,26,27,28,29]. This discrepancy may be attributed to differences in the study populations, as well as in the severity and heterogeneity of the instruments used to measure OHRQoL. Indeed, the use of different validated questionnaires, which are characterized by domains, scoring systems, and scoring directions that may not be directly comparable, may limit the ability to integrate, fully interpret, and compare the reported findings.

Therefore, the primary aim of the present umbrella review was to evaluate the overall and domain-specific OHRQoL impacts among pediatric subjects with MIH and among their caregivers/parents.

Secondary aims were to evaluate the influence of MIH severity and/or lesion extent/distribution pattern and age and/or dentition status on overall- and domain-specific OHQoL outcomes, and to assess the impact of different dental treatments on OHRQoL.

## 2. Materials and Methods

### 2.1. Study Protocol

The study protocol was developed following the Preferred Reporting Items for Systematic Reviews and Meta-analyses (PRISMA) guidelines (Supplementary File S1) [30] and registered on the International Prospective Register of Systematic Reviews (PROSPERO) under the registration number CRD420261445745. The study protocol was developed prior to conducting the literature search, data extraction, and analysis.

This umbrella review addressed the following questions: “What is the overall oral health-related quality of life (OHRQoL) among pediatric subjects with molar incisor hypomineralization (MIH)? What is the impact of MIH on the various domains of OHRQoL in pediatric subjects, considering both pediatric patients’ and their parents’/caregivers’ perceptions? How does the severity and/or extent/distribution pattern of MIH, along with age/dentition status, influence OHRQoL in pediatric patients, both overall and across specific domains? What is the impact of different treatment strategies on the overall and domain-specific oral health-related quality of life in pediatric patients with MIH?”

The formulation of the research questions, the development of the search strategy, and the determination of study selection criteria were guided by the PEO model [31], as detailed below:

P—Population: Pediatric subjects (≤18 years of age);
E—Exposure: The presence of molar incisor hypomineralization (MIH) diagnosed according to validated diagnostic criteria or internationally accepted classification systems (e.g., EAPD, Ghanim, DDE Index) [1,32,33];
O—Outcome(s):
Primary outcome(s): Overall and domain-specific OHRQoL (encompassing oral, functional, psychological, and social domains), measured through validated child- and/or parent-reported outcome measures;
Secondary outcome(s): The influence of MIH severity and/or lesion extent/distribution pattern and age/dentition status on both overall and domain-specific OHRQoL, as well as the impact of different therapeutic interventions on both overall and domain-specific OHRQoL, by assessing pre- versus post-treatment changes.

### 2.2. Search Strategy

Two independent reviewers (M.P. and G.S.) performed an electronic search to identify systematic reviews, both with and without meta-analysis, up to 28 April 2026.

The search involved the following databases and registers and applying filters according to availability:

- PubMed/MEDLINE: Article type “Systematic Review” and “Meta-analysis”;
- Scopus (search within: Title, abstract, keywords): Document type “Review”;
- Web of Science (search within: all fields): Document type “Review article”;
- PROSPERO register: Review status “Completed”;
- The Cochrane Library (search within: Title, abstract, keywords).

No date restrictions were applied.

The following keywords combined with Boolean operators were used for each database:

(“quality of life” OR “oral health related quality of life” OR QoL OR “OHR-QoL”) AND (MIH OR “molar incisor hypomineralization”) AND (children OR adolescent OR infant OR childhood OR young).

### 2.3. Study Selection and Eligibility Criteria

After establishing the eligibility criteria, two independent reviewers (M.P. and G.D.B.) performed the study selection process. Any disagreements were addressed through discussion, with the involvement of a third reviewer (M.d.C.V.-V.), if necessary, to reach a consensus.

Titles and abstracts from the electronic search were screened to remove duplicates and irrelevant records. If titles or abstracts were unclear, the full text was reviewed before exclusion. When full texts were unavailable, corresponding study authors were contacted to obtain them.

A manual search of the reference lists in the included studies was also performed to identify any additional eligible records.

References from the selected studies were exported and managed with Mendeley Reference Manager version 2.120.3, copyright Elsevier Ltd. (Amsterdam, The Netherlands).

Inclusion criteria were as follows: systematic reviews, with or without meta-analysis, published in the English language, without any restrictions concerning publication date, sample size, gender, or country. Eligible studies were human studies evaluating OHRQoL in pediatric subjects (≤18 years of age), assessed through any validated child- or parent-reported outcome measure, diagnosed with molar incisor hypomineralization (MIH) according to any recognized diagnostic criteria or classification system, with no restrictions on MIH severity, lesion extent/distribution pattern, or dentition status. Systematic reviews were considered eligible when assessing OHRQoL in both untreated subjects and in those before and/or after any therapeutic intervention for MIH.

Exclusion criteria included non-systematic reviews; studies published in languages other than English; studies not conducted in humans; systematic reviews conducted on adult subjects (>18 years of age), or in which it was not possible to extract data on pediatric subjects (≤18 years of age); systematic reviews evaluating enamel defects other than MIH, or those where MIH was not diagnosed using recognized diagnostic criteria; and systematic reviews that did not assess OHRQoL or did not employ validated child- or parent-reported outcome measures.

**Figure 1:**
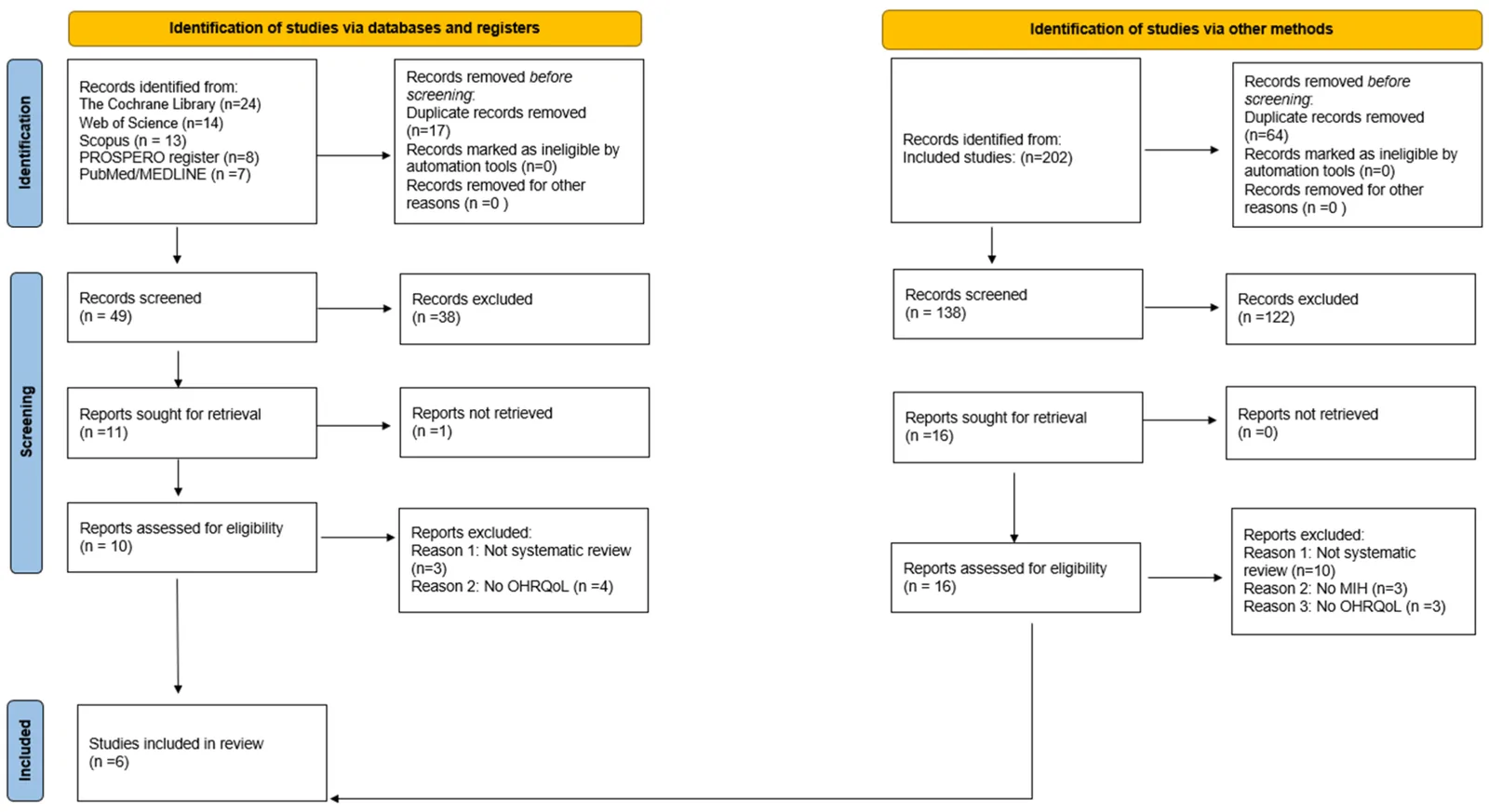
PRISMA flowchart.

The study selection and eligibility criteria processes are depicted in Figure 1.

### 2.4. Data Extraction and Collection

Data were extracted and collected by three independent reviewers (M.P.; E.F.; M.S.), using a standardized data extraction form based on the established model for randomized and non-randomized intervention reviews. Any discrepancies were resolved by consensus or, if necessary, by consulting a fourth reviewer (G.D.B.).

The following data were extracted and collected from each systematic review, regardless of whether meta-analysis was performed:

- Study characteristics: first author, year of publication, journal, reference, study design and number of studies included, meta-analysis (if performed), and quality assessment;
- Population characteristics: sample size, mean age/age range, gender ratio (M/F), country, and dentition status;
- Exposure: MIH classification system, prevalence, severity, extent, and treatment (if any);
- Outcomes: OHRQoL tool, respondents (child, parents), and mean overall OHRQoL values.

Only data that adhered to the established eligibility criteria were included in the extraction process. Consequently, data not about pediatric populations, data concerning enamel defects other than MIH, or data derived from non-validated OHRQoL instruments were excluded from the extraction and collection processes. Systematic reviews that included both pediatric and adult populations or evaluated multiple dental conditions alongside MIH were considered eligible; however, only the data specifically related to pediatric populations diagnosed with MIH were extracted, while data concerning adult populations or other enamel pathologies/conditions were excluded.

### 2.5. Data Synthesis

Data from the systematic reviews (with or without meta-analysis) included in the present umbrella review were descriptively synthesized through Microsoft Excel Software 2019 (Microsoft Corporation, Redmond, WA, USA). Due to the pronounced methodological heterogeneity in OHRQoL questionnaires and data reporting formats across the selected literature, statistical pooling of effect sizes or formal quantitative meta-analysis was not performed. Instead, qualitative and descriptive comparison was achieved by converting the extracted mean scores into a descriptive normalized percentage impact index. To achieve a standardized qualitative synthesis, mean scores were harmonized using the Percent of Maximum Possible (POMP) methodology, which normalizes scores onto a descriptive scale ranging from 0 to 100%, preserving the absolute distances between the original values and rendering different metrics comparable for descriptive aggregation [34].

The data synthesis was focused and organized around these goals:

- To assess overall and domain-specific OHRQoL in pediatric subjects (≤18 years of age) with MIH;
- To assess overall and domain-specific OHRQoL, sorted by children and parents, and both overall values;
- To assess and compare overall and domain-specific OHRQoL between child self-reported and parental perception;
- To evaluate the impact of MIH severity and/or extent/distribution pattern on overall and domain-specific OHRQoL;
- To assess the impact of age/dentition status on overall and domain-specific OHRQoL;
- To assess the impact of the different dental treatments on overall and domain-specific OHRQoL.

#### 2.5.1. OHRQoL Scale Normalization

To address the lack of metric consistency from the use of multiple scales for assessing OHRQoL, including the different versions of the Child Perceptions Questionnaire (CPQ 8–10 and CPQ 11–14), the Parental–Caregiver Perception Questionnaire (P-CPQ), and the Oral Health Impact Profile (OHIP-14), a linear normalization calculation of the scores was performed. The scales and questionnaires identified have mathematical structures and scoring procedures that are inherently incomparable, characterized by both variable numbers of items and different theoretical maximum scores for each domain. To enable descriptive comparisons across different age cohorts, respondents, and domains, the original weighted mean scores were converted into a descriptive percentage impact index. Metric standardization was performed by applying two different mathematical proportionality models, differentiated based on the nature of the measurement instrument: for questionnaires belonging to the CPQ family (CPQ 8–10, CPQ 11–14), the P-CPQ, and the OHIP-14, where a higher mathematical score reflects worsening of QoL, the conversion was performed using the following formula:

$$\mathrm{Percentage}\;\mathrm{Impact}\;\mathrm{Index}\;\left(\mathrm{PII}\right)\left(\%\right)=\left(\frac{\mathrm{Weighted}\;\mathrm{Mean}\;\mathrm{Domain}\;\mathrm{Score}}{\mathrm{Theoretical}\;\mathrm{Maximum}\;\mathrm{Value}\;\mathrm{of}\;\mathrm{the}\;\mathrm{Domain}}\times 100\right)$$

On the contrary, the methodological discrepancy for the Child Oral Health Impact Profile (C-OHIP) scale is that it uses an opposite scoring direction to the previous ones, whereby higher scores indicate a better quality of life and a lesser impact of the condition. Therefore, to maintain the conceptual consistency of the percentage impact index and allow for direct comparison among all scales, the normalization formula for the C-OHIP domains was modified mathematically by inverting the weighted mean score as follows:

$$\mathrm{Percentage}\;\mathrm{Impact}\;\mathrm{Index}\;\left(\mathrm{PII}\right)\left(\%\right)=\left(1-\frac{\mathrm{Weighted}\;\mathrm{Mean}\;\mathrm{Domain}\;\mathrm{Score}}{\mathrm{Theoretical}\;\mathrm{Maximum}\;\mathrm{Value}\;\mathrm{of}\;\mathrm{the}\;\mathrm{Domain}}\times 100\right)$$

The percentual index thus obtained expresses the impact of disease in a standardized range between 0% and 100%. This approach follows the Percent of Maximum Possible (POMP) score, which is a method used to normalize heterogeneous multi-item scales into a single communicative percentage metric (0–100%), intended exclusively for descriptive comparative purposes [34].

Conceptually, the PII% represents the proportion of the maximum theoretical impairment score measurable on a given instrument (where 0% reflects an optimal, uncompromised OHRQoL and 100% the maximum possible adverse impact on OHRQoL). Therefore, a score on this continuum indicates the relative positioning of perceived impairment within the theoretical scale range, and should not be interpreted as meaning that MIH may cause an absolute/literal percentage reduction in OHRQoL.

#### 2.5.2. Calculation of Overall and Subgroup OHRQoL Values

After converting the average scores from each study into their respective descriptive percentage impact index (on a standardized scale ranging from 0% to 100%), the data were aggregated to provide three cumulative summary domains for each macro-area: overall child impact percentage (relating solely to the scales completed by children/adolescents), overall parental impact percentage (relating to the scales completed by parents/caregivers), and overall impact percentage (an absolute summary that combines the perceptions of children and parents). For each domain, the overall values were calculated using a weighted-average model based on each scale’s sample size. The formula applied to calculate the overall domain impact index (and similarly adapted for the child and parental assessment, taking into account their respective sample subgroups) was as follows:

$$\mathrm{Overall}\;\mathrm{domain}\;\mathrm{impact}\;\mathrm{index}\;(\%)=\sum \frac{\left(\mathrm{PII}\times n\right)}{N}$$

where ∑(*PII* × *n*) represents the sum of the products between the percentage impact index of each individual scale and its corresponding sample size (*n*), and *N* is the total sample size obtained by summing all participants included within that specific macro-dimension.

In addition, overall descriptive OHRQoL impact percentages, sorted into overall child OHRQoL impact and parent-reported OHRQoL impact, were calculated as the arithmetic mean of the respective four normalized domain-specific impact percentages (oral, functional, psychological, and social domains) for descriptive comparative purposes.

Furthermore, regarding the assessment of OHRQoL percentage impact scores by age/dentition status (e.g., early mixed dentition versus late mixed/permanent dentition), in cases where systematic reviews reported heterogeneous or broad age ranges (e.g., 6–16 years), the corresponding data, although systematically extracted at the single-domain level, were excluded from the subgroup assessment due to the impossibility of setting the ranges into precise dentition stages. Similarly, for OHRQoL assessment tools, instruments for which primary studies provided only narrative findings or lacked numerical data convertible to the 0–100% scale under the described normalization process (e.g., ECHOIS and C-OIDP) were retained exclusively for qualitative synthesis and excluded from domain-specific impact percentage analyses.

#### 2.5.3. Treatment Impact on OHRQoL Calculation

To evaluate the impact of dental treatments for MIH on OHRQoL, scale normalization and the overall domain impact index described above were applied to pre- and post-treatment assessments as descriptive comparative indices. The dental treatments were categorized as follows: preventive/desensitizing treatment; microabrasion/infiltration-based treatment; restorative treatment; and combined treatment. In cases where a tool (specifically, the OHIP-14) provided separate scores for subdomains within the same macro-area (psychological discomfort and psychological disability; social disability and handicap), their respective means were summed prior to normalization, yielding a single score for each domain. For each treatment domain, according to each scale’s directional criteria (including the inverted formula for C-OHIP), the overall pre- and post-treatment descriptive percentage impact index was calculated, considering the sample size. Finally, the overall pre- and post-treatment descriptive percentage index of OHRQoL was calculated. The calculation of the descriptive net therapeutic gain was obtained by using the percentage impact improvement (Δ) as follows:

Δ = Percentage Impact Index*_Pre-treatment_* − Percentage Impact Index*_Post-treatment_*

The descriptive net therapeutic gain (Δ) reflects the mathematical, descriptive difference between baseline and post-treatment percentage impact scores, serving as a comparative indicator of overall and domain-specific OHRQoL impact variations rather than an absolute or fixed rate of clinical improvement across interventions.

### 2.6. Quality Assessment and Overlap Management

The systematic reviews (with or without meta-analysis) were qualitatively assessed by two independent reviewers (M.P., G.S.), through the Assessing the Methodological Quality of Systematic Reviews 2 (AMSTAR) tool, available online at https://amstar.ca/ (accessed on 4 July 2026) for the quality valuation of systematic reviews of randomized and non-randomized studies. In the event of any disagreement during the quality assessment process, a third reviewer (G.D.B.) was consulted to reach consensus.

The corrected covered area (CCA) was calculated to evaluate the overlap of primary studies among the included systematic reviews.

## 3. Results

### 3.1. Study Selection

The electronic search identified 66 records: 24 in the Cochrane Library, 14 in Web of Science, 13 in Scopus, 8 in the PROSPERO register, and 7 in PubMed/MEDLINE. A total of 17 duplicate records were removed prior to screening. Of the remaining 49 records, 38 were excluded, as they did not pertain to the study topic. One record was not retrieved; the study authors were contacted, but no response was obtained, and the record was excluded.

The 10 remaining records were assessed for eligibility, and 4 were excluded, specifically because 3 were not systematic reviews and 1 did not assess OHRQoL.

Before the manual search, a total of 6 systematic reviews [35,36,37,38,39,40] were included from the electronic search.

From the manual search of the reference lists of the included systematic reviews, 202 additional records were found. A total of 64 duplicates were removed, and after which 122 records were excluded as they did not pertain to the study topic. The remaining 16 records were evaluated for eligibility, but full-text screening excluded all of them, specifically for the following reasons: 10 were not systematic reviews; 3 did not assess MIH; and 3 did not assess OHRQoL.

Therefore, a total of six systematic reviews [35,36,37,38,39,40] were included in the present umbrella review (Figure 1).

### 3.2. Study Characteristics and Qualitative Synthesis

Data extracted from the six systematic reviews (with or without meta-analysis) [35,36,37,38,39,40] included in the present systematic review are presented in Supplementary File S2, which details study characteristics, population, exposure, and outcomes.

Among the systematic reviews included in this umbrella review, four included a meta-analysis and two did not. The study designs of the primary studies included in the reviews were as follows: 35 cross-sectional studies, 7 case–control studies, 6 observational studies, 5 non-randomized clinical trials, and 1 randomized controlled trial (RCT).

The total study population across the primary studies in the systematic reviews comprised 24,404 pediatric subjects; the gender ratio was reported for a subsample of 12,174 subjects, including 7135 males and 5039 females. Study participants came from the following countries: Brazil (*n* = 8303); Iran (*n* = 5014); Nigeria (*n* = 4265); Germany (*n* = 2841); Mexico (*n* = 1644); Australia (*n* = 1320); Belgium (*n* = 290); Columbia (*n* = 264); the UK (*n* = 196); China (*n* = 110); Turkey (*n* = 80); and Austria (*n* = 77).

**Figure 2:**
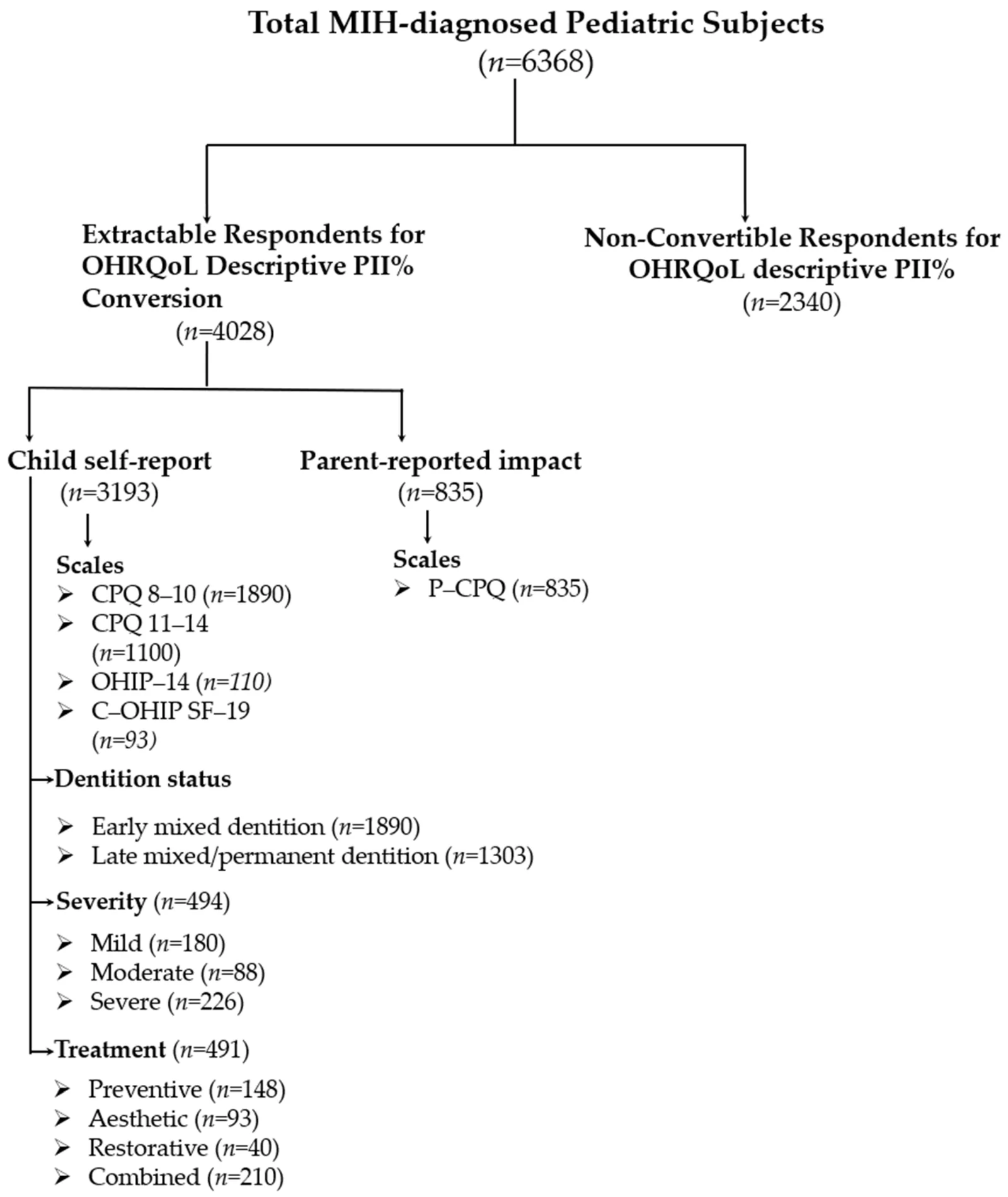
Study characteristics. Abbreviations: MIH, “molar incisor hypomineralization”; OHRQoL, “oral health-related quality of life”; PII%, “percentage impact index”; %, “percentage”; CPQ 8–10, “Child Perceptions Questionnaire for Children Aged 8–10 Years”; CPQ 11–14, “Child Perceptions Questionnaire for Adolescents Aged 11–14 Years”; OHIP-14, “Oral Health Impact Profile—14-item Version”; C-OHIP SF-19, “Child Oral Health Impact Profile—Short Form 19”; P-CPQ, “Parental–Caregiver Perception Questionnaire”.

Based on the inclusion criteria of this umbrella review, 6368 pediatric subjects were diagnosed with MIH (Figure 2), with a reported mean age of 9.78 years (*n* = 491) and an age range of 6 to 18 years. Among MIH-affected pediatric subjects, country of provenance was reported for 491 subjects as follows: Germany (*n* = 210); China (*n* = 110); the UK (*n* = 93); Turkey (*n* = 40); and Austria (*n* = 38).

The reported gender ratio among subjects diagnosed with MIH was 233 males to 258 females (1:1.10). MIH diagnoses were made using the following classification systems: the European Academy of Pediatric Dentistry (EAPD) (*n* = 5395); the Developmental Defects of Enamel (DDE) Index (*n* = 685); Ghanim scoring system (*n* = 194); and the Molar Incisor Hypomineralization Treatment Need Index (MIH-TIN) (*n* = 94).

In pediatric patients diagnosed with MIH, dentition status was reported in 6263 subjects as follows: early mixed dentition (*n* = 2348); late mixed/permanent dentition (*n* = 1747). In 2168 patients, the dentition status was not defined, falling between mixed and permanent. For these pediatric subjects, it was not possible to precisely range the dentition status.

MIH severity across the total MIH-diagnosed subjects was defined for 4635 subjects as follows: mild (*n* = 2391); moderate (*n* = 193); moderate/severe (*n* = 449); severe (*n* = 1602); and no defined severity (*n* = 1733). MIH extent/distribution pattern, including the number of teeth affected and lesion extension, was not assessed in any of the included systematic reviews.

Across the included systematic reviews, the following scales/tools were used to assess OHRQoL, with both child and parent respondents: CPQ 8–10 (*n* = 3161); Parental–Caregiver Perception Questionnaire (P-CPQ) (*n* = 1197); CPQ 11–14 (*n* = 1041); Child Oral Health Impact Profile-Short Form 19 (C-OHIP SF-19) (*n* = 310); Early Childhood Oral Health Impact Scale (ECHOIS) (*n* = 124); Oral Health Impact Profile-14 (OHIP-14) (*n* = 110); Child Oral Impacts on Daily Performances (C-OIDP) (*n* = 25). While all scales/tools were included in the qualitative synthesis (Supplementary File S2), instruments such as ECHOIS and C-OIDP, as well as narrative-only datasets, could not be converted into standardized 0–100% domain metrics, and the corresponding respondents (*n* = 2340) were therefore not included in the OHRQoL overall and domain-specific analyses.

From the total pool of MIH-affected individuals, a total of 4028 respondents contributed extractable domain scores convertible into the descriptive percentage impact index, comprising 3193 child respondents providing self-reported data through the CPQ 8–10 (*n* = 1890), CPQ 11–14 (*n* = 1100), OHIP-14 (*n* = 110), and C-OHIP SF-19 (*n* = 93), and 835 parent/caregiver respondents providing proxy-reported data through P-CPQ.

Among the 3193 child respondents included in the descriptive percentage impact index analysis for overall and domain-specific OHRQoL, *n* = 1890 were in the early mixed dentition stage (assessed through CPQ 8–10) and 1303 were in the late mixed/permanent dentition stage (assessed via CPQ 11–14, *n* = 1100; C-OHIP SF-19, *n* = 93; OHIP-14, *n* = 110).

Additionally, OHRQoL values stratified by severity were available and convertible into the descriptive percentage impact index in an exploratory subsample of 494 child respondents aged 8–10 years, distributed into mild (*n* = 180), moderate (*n* = 88), and severe (*n* = 226) forms.

The mean values were reported for the following scales/tools: the mean CPQ 8–10 was 19.81 assessed in 1785 pediatric subjects; the mean CPQ 11–14 was 14.28 (*n* = 846); the mean P-CPQ was 12.26 (*n* = 835); the mean OHIP-14 was 17.61 ± 4.22 (*n* = 110); the mean C-OHIP SF-19 was 47.00 ± 9.29 (*n* = 93).

The reported dental treatments for MIH in 491 pediatric subjects were as follows: preventive/desensitizing treatment (*n* = 148), which comprised pit and fissure sealants, both resin-based and glass ionomer, and an Er:YAG laser with topical desensitizer applications; minimally invasive esthetic treatment (*n* = 93), which comprised enamel microabrasion, resin infiltration, tooth whitening, and direct composite restorations; restorative treatment (*n* = 40), with caries removal and glass hybrid restorations; and combined treatment strategies (*n* = 210) which comprised preventive, minimally invasive esthetic, restorative, endodontic, prosthodontic and extraction procedures according to MIH severity.

#### 3.2.1. Domain-Specific Impact on OHRQoL

The domain-specific impact on OHRQoL, divided into oral, functional, psychological, and social domains across the various scales, along with the corresponding mean scores and impact percentages, the overall respondent-dependent impact (child impact, parent-reported impact), and the total domain-specific and overall OHRQOL impact are presented in Table 1.

##### Oral Domain

The oral domain, which comprised oral symptoms and physical pain, was assessed in 4028 respondents, resulting in the following domain impact percentage, according to each scale used: 32.9% (*n* = 1890) in the CPQ 8–10 [35,38,40]; 21.0% (*n* = 1100) in the CPQ 11–14 [37,38,40]; 43.7% (*n* = 93) in the C-OHIP SF19 [38]; 50.5% (*n* = 110) in the OHIP-14 [38]; and 13.86% (*n* = 835) in the P-CPQ [37,40]. Specifically, the overall oral domain child impact percentage was 29.72%, and the overall oral domain parent-reported impact percentage was 13.86%.

Therefore, the overall impact on the oral domain, across both children and parents, was 26.43%.

##### Functional Domain

The functional domain was assessed in 3918 respondents, resulting in the following domain impact percentage, according to each scale used: 29.1% (*n* = 1890) in the CPQ 8–10 [35,38,40]; 9.2% (*n* = 1100) in the CPQ 11–14 [37,38,40]; 17.0% (*n* = 93) in the C-OHIP SF19 [38]; and 11.62% (*n* = 835) in the P-CPQ [37,40]. Specifically, the overall functional domain child impact percentage was 21.63%, and the overall functional domain parent-reported impact percentage was 11.62%.

Therefore, the overall impact on the functional domain, across both children and parents, was 19.50%.

##### Psychological Domain

The psychological domain, which comprised emotional well-being, psychological discomfort, and psychological disability, was assessed in 3919 respondents, resulting in the following domain impact percentage, according to each scale used: 28.9% (*n* = 1890) in the CPQ 8–10 [35,38,40]; 8.6% (*n* = 1100) in the CPQ 11–14 [37,38,40]; 43.9% (*n* = 93) in the C-OHIP SF19 [38]; 42.9% (*n* = 110) and 24.5% (*n* = 110) for psychological discomfort and psychological disability, respectively, in the OHIP-14 [38]; and 10.72% (*n* = 726) in P-CPQ [37,40]. Specifically, the overall psychological domain child impact percentage was 22.42%, and the overall psychological domain parent impact percentage was 10.72%.

Therefore, the overall impact on the psychological domain, across both children and parents, was 20.25%.

##### Social Domain

The social domain, which comprised social well-being, social disability, and handicap, was assessed in 3919 respondents, resulting in the following domain impact percentage, according to each scale used: 16.2% (*n* = 1890) in the CPQ 8–10 [35,38,40]; 4.3% (*n* = 1100) in the CPQ 11–14 [37,38,40]; 43.9% (*n* = 93) in the C-OHIP SF19 [38]; 24.1% (*n* = 110) and 15.6% (*n* = 110) for social disability and handicap, respectively, in the OHIP-14 [38]; and 5.12% (*n* = 726) in the P-CPQ [37,40]. Specifically, the overall social domain child impact percentage was 13.26%, and the overall social domain parent-reported impact percentage was 5.12%.

Therefore, the overall impact on the social domain, across both children and parents, was 11.75%.

**Figure 3:**
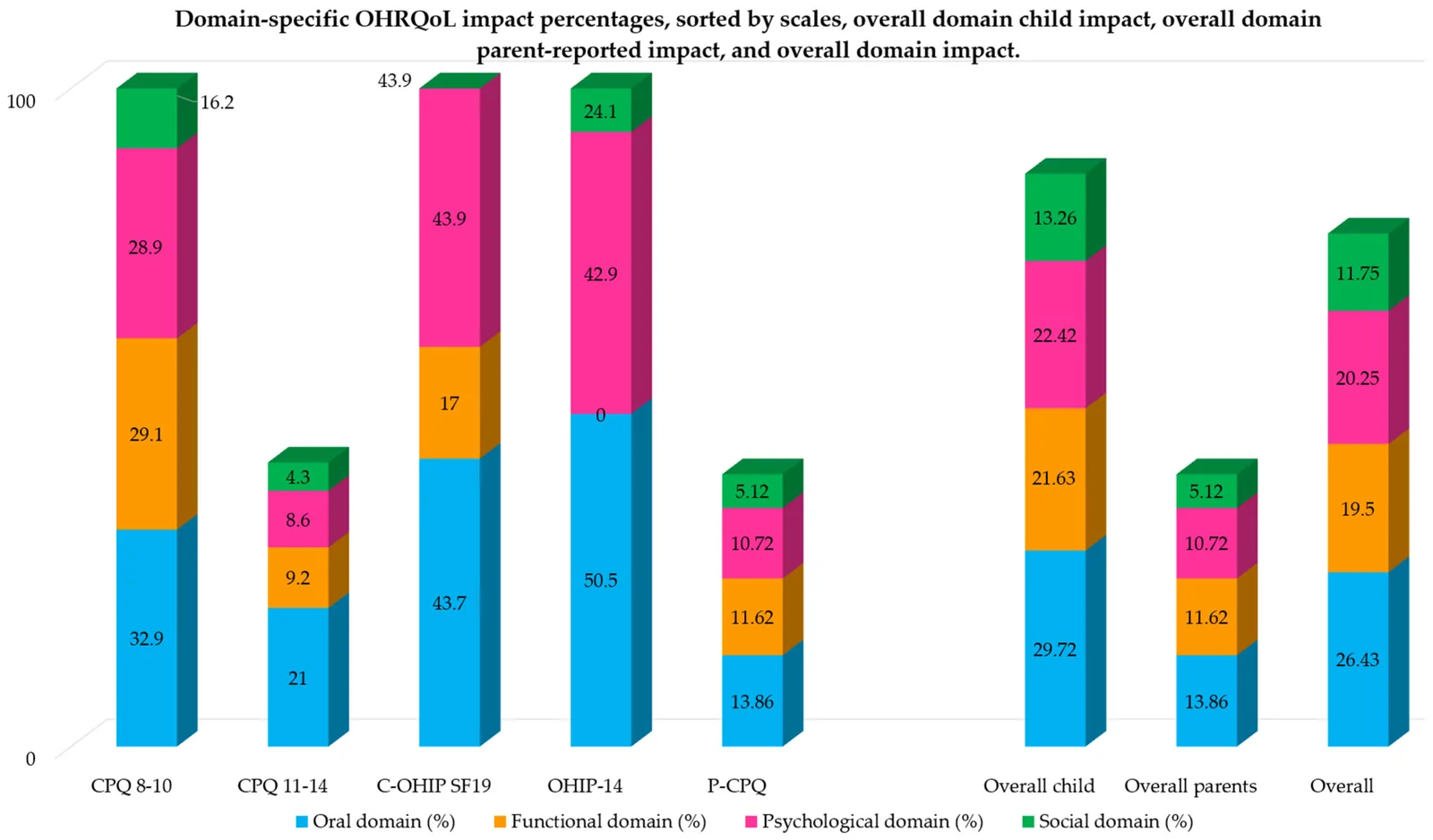
The domain-specific and overall impact percentages, sorted by scales and respondents.

Figure 3 shows the domain-specific OHRQoL impact percentages, sorted by scales, the overall domain child impact, the overall domain parent-reported impact, and the overall domain impact.

#### 3.2.2. Overall OHRQoL in Children and Parents

The reported overall child OHRQoL impact, comprising oral, functional, psychological, and social domains, was 21.76%.

The reported overall parent OHRQoL impact, comprising oral, functional, psychological, and social domains, was 10.33%.

#### 3.2.3. Domain-Specific and Overall OHRQoL and MIH Severity and/or Extent/Distribution Pattern

In the present umbrella review, OHRQoL, sorted by severity, was assessed in a subsample of 494 pediatric subjects aged 8–10 years, in the early mixed dentition stage, using the CPQ 8–10 [35].

Supplementary File S3 presents the specific OHRQoL domains assessed across the scales, stratified by assessed severity levels with mean values and the percentage index.

Among the 494 pediatric subjects, MIH severity was distributed as follows: mild (*n* = 180), moderate (*n* = 88), and severe (*n* = 226) [35].

The oral domain impact percentage across the investigated population was as follows: 19.00% in the mild category, 18.00% in the moderate category, and 32.00% in the severe category [35].

The functional domain impact percentage across the investigated population was as follows: 12.70% in mild, 12.50% in moderate, and 24.50% in severe [35].

The psychological domain impact percentage across the investigated population was as follows: 16.10% in mild; 13.50% in moderate; and 25.00% in severe [35].

The social domain impact percentage across the investigated population was as follows: 8.90% in mild; 1.25% in moderate; and 3.30% in severe [35].

The overall impact of OHRQoL among the subjects with mild MIH was 14.18%, 11.31% for moderate, and 21.20% for severe [35].

None of the included systematic reviews assessed OHRQoL sorted by the number of teeth affected and/or lesion extension/distribution pattern.

#### 3.2.4. Domain-Specific and Overall OHRQoL and Dentition Status

The domain-specific and overall impact on OHRQoL, sorted by dentition status across the various scales, along with the corresponding mean scores and impact percentages, is presented in Table 2.

##### Early Mixed Dentition Status

OHRQoL was assessed in 1890 children with early mixed dentition status, comprising children aged 8 to 10 years [35,38,40], through the CPQ 8–10.

The overall impact percentages were 32.9% on the oral domain, 29.1% on the functional domain, 28.9% on the psychological domain, and 16.2% on the social domain [35,38,40].

Therefore, the overall child impact on OHRQoL, comprising oral, functional, psychological, and social domains, was 26.77% in children with early mixed dentition status [35,38,40].

##### Late Mixed/Permanent Dentition Status

Regarding late mixed/permanent dentition status, OHRQoL was assessed in 1303 pediatric subjects aged 11 to 14 years using the following scales: CPQ 11–14 (*n* = 1100), C-OHIP SF-19 (*n* = 93), and OHIP-14 (*n* = 110) [37,38,40].

The oral domain, which comprised oral symptoms and physical pain, resulted in the following impact percentages for each scale/tool used: 21.0% in the CPQ 11–14; 43.7% in the C-OHIP SF-19; and 50.5% in the OHIP-14 [37,38,40].

Therefore, the overall domain-specific child impact on the oral domain was 25.11% [37,38,40].

The functional domain impact percentages for each scale/tool were as follows: 9.2% in the CPQ 11–14 and 17.0% in the C-OHIP SF-19 [37,38,40].

Therefore, the overall domain-specific child impact on the functional domain was 9.81%.

The psychological domain, which comprised emotional well-being, psychological discomfort, and psychological disability, resulted in the following impact percentages for each scale/tool used: 8.6% in the CPQ 11–14; 43.9% in the C-OHIP SF-19, and 42.9% and 24.5% for psychological discomfort and disability, respectively, in the OHIP-14 [37,38,40].

Therefore, the overall domain-specific child impact on the psychological domain was of 14.02%.

The social domain, which comprised social well-being, social disability and handicap, resulted in the following impact percentages for each scale/tool used: 4.3% in the CPQ 11–14; 43.9% in the C-OHIP SF-19; and 24.1% and 15.6% for social disability and handicap, respectively, in the OHIP-14.

Therefore, the overall domain-specific child impact on the social domain was 8.80%.

The overall child impact on OHRQoL, comprising oral, functional, psychological, and social domains, was 14.43% in children with late mixed/permanent dentition status.

#### 3.2.5. Dental Treatment: Improvement in Domain-Specific and Overall OHRQoL

One systematic review [38] analyzed the impact of treatment on OHRQoL. The dental treatments performed on 491 subjects with MIH were as follows: preventive/desensitizing treatment (*n* = 148), which comprised pit and fissure sealants, both resin-based and glass ionomer, and an Er:YAG laser with topical desensitizer applications; minimally invasive esthetic treatment (*n* = 93), which comprised enamel microabrasion, resin infiltration, tooth whitening, and direct composite restorations; restorative treatment (*n* = 40), with caries removal and glass hybrid restorations; and combined treatment (*n* = 210) which comprised preventive, minimally invasive esthetic, restorative, endodontic, prosthodontic and extraction procedures according to MIH severity.

The reported follow-up was as follows: 3–5 months for preventive/desensitizing treatment (*n* = 148); 1 month for minimally invasive esthetic treatment (*n* = 93); 6 months for restorative treatment (*n* = 40); and no defined follow-up in combined treatment (*n* = 210) [38].

Table 3 details the impact on specific OHRQoL domains for pre-treatment, post-treatment, and improvement, expressed as percentages.

In MIH subjects treated with preventive/desensitizing treatment (*n* = 148), the greatest improvement was in the oral domain (36.46% vs. 21.75% in the psychological domain, 19.50% in the functional domain, and 10.84% in the social domain) [38].

In MIH subjects treated with minimally invasive esthetic treatment (*n* = 93), the psychological and social domains showed the greatest improvement (18.65% in both domains vs. 14.45% in the oral domain and 5.50% in the functional domain) [38].

In MIH subjects treated with restorative treatment (*n* = 40), the psychological domain showed the greatest improvement (24.14% vs. 23.66% in the oral domain, 10.69% in the functional domain, and 6.50% in the social domain) [38].

In MIH subjects treated with combined treatment (*n* = 210), the oral domain showed the greatest improvement (16.00% vs. 15.45% in the psychological, 14.10% in the functional, and 3.05% in the social domain) [38].

The overall impact on OHRQoL in MIH subjects treated with preventive/desensitizing treatment (*n* = 148) was 28.09% at baseline and 5.95% after treatment, representing an improvement of 22.14% in OHRQoL [38].

The overall impact on OHRQoL in MIH subjects treated with minimally invasive esthetic treatment (*n* = 93) was 37.10% at baseline and 22.79% after treatment, representing a 14.31% improvement [38].

The overall impact on OHRQoL in MIH subjects treated with restorative treatment (*n* = 40) was 25.23% at baseline and 8.98% after treatment, representing a 16.25% improvement in OHRQoL [38].

The overall impact on OHRQoL in MIH subjects treated with combined treatment (*n* = 210) was 21.37% at baseline and 9.22% after treatment, representing a 12.15% improvement in OHRQoL.

**Figure 4:**
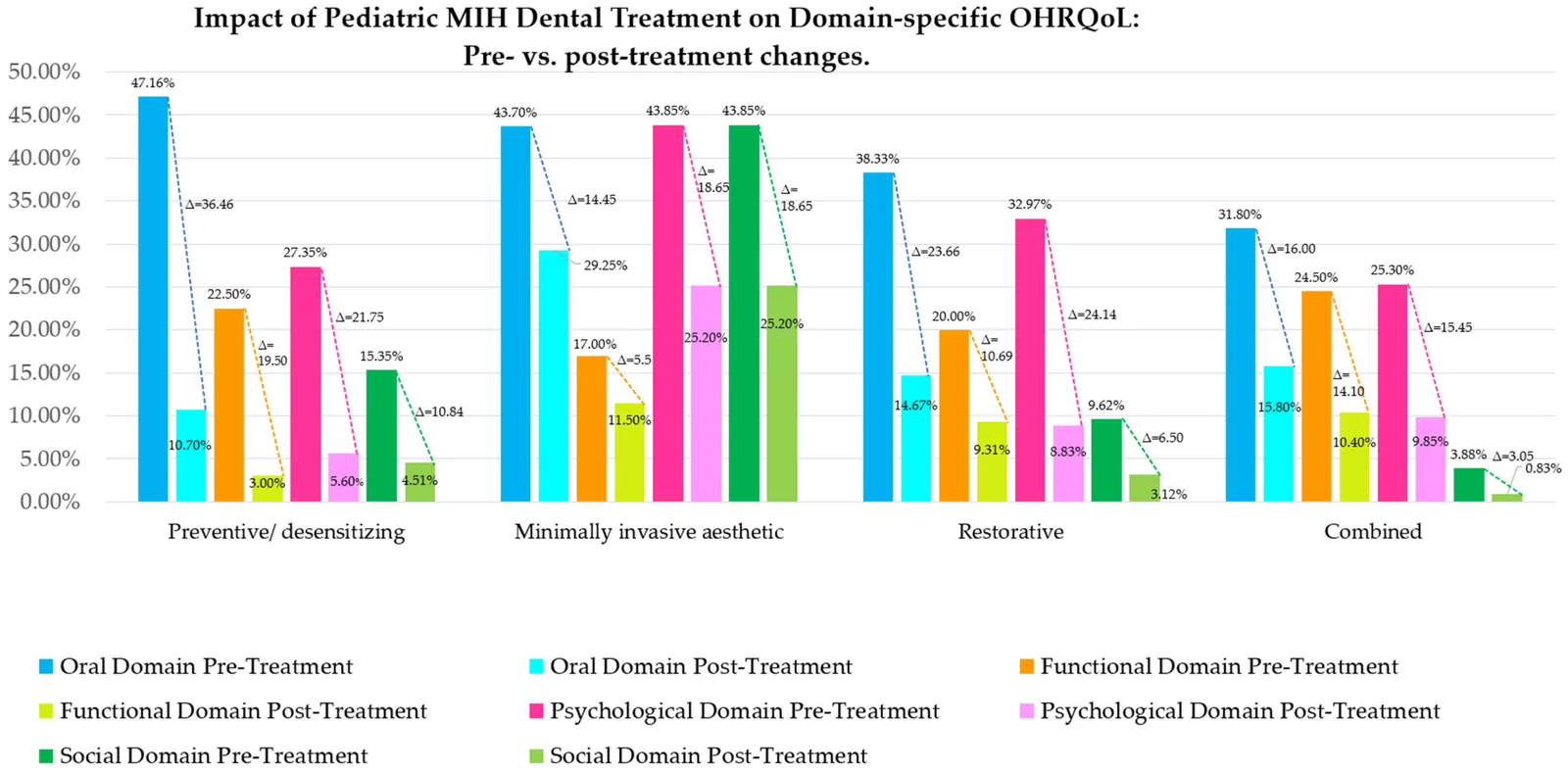
The impact of dental treatment on MIH-affected pediatric individuals sorted by treatment type (preventive/desensitizing, minimally invasive esthetic, restorative and combined treatment) and domain-specific impact percentages before and after treatment and the related net improvement (Δ) percentages, shown as a dashed line representing the difference between the pre- and post-treatment impact percentages.

Figure 4 depicts the impact of pediatric MIH dental treatment on domain-specific OHRQoL pre- and post-treatment changes, as well as the improvement (Δ).

### 3.3. Quality Assessment and Overlap of Primary Studies

The quality of the six included systematic reviews [35,36,37,38,39,40] was assessed with AMSTAR-2 for each domain (Supplementary File S4). The overall judgment resulted in zero high-quality studies (0.00%), two moderate-quality studies (33.33%) [38,39], two low-quality studies (33.33%) [35,37], and two critically low-quality studies (33.33%) [36,40].

The CCA was 31.43% (21 unique primary studies, 54 total primary study inclusions, and 6 systematic reviews analyzed), calculated using the methodology of Pieper et al. [41]. According to the overlap grading by Pieper et al. [41] (0–5% “slight”; 6–10% “moderate”; 11–15% “high”; >15% “very high”), a CCA of 31.43% indicates a “very high” primary study overlap.

## 4. Discussion

The impact of MIH on children’s oral health may extend beyond the clinical manifestations of enamel hypomineralization, involving different aspects of everyday life and well-being. Symptoms such as hypersensitivity and pain, together with functional difficulties and esthetic concerns, may be experienced differently according to the child’s developmental stage and individual perception, while also being interpreted differently by parents and caregivers.

From this perspective, OHRQoL may provide a broader understanding of the burden associated with MIH, allowing its clinical manifestations to be considered in relation to the child’s subjective experience and to the different factors that may shape it.

Therefore, the primary aim of the present umbrella review was to evaluate the overall and domain-specific impacts on OHRQoL among pediatric subjects with MIH, as well as among caregivers/parents. Secondary aims were to evaluate the influence of MIH severity and/or lesion extent/distribution pattern and age and/or dentition status on overall and domain-specific OHQoL outcomes, and to assess the impact of different dental treatments on OHQoL.

### 4.1. Overall and Domain-Specific OHRQoL: Children’s Self-Reported and Parent/Caregiver Proxy-Reported Impact

The present umbrella review showed a relevant and multidimensional impact among the pediatric population assessed, yet revealed a marked and consistent discrepancy in respondents’ perceptions. However, when interpreting the findings of the present umbrella review, specifically overall and domain-specific OHRQoL among children and parents/caregivers, it should be noted that the descriptive impact percentages reported represent author-derived descriptive indices resulting from scale normalization rather than formal statistical pooled effect sizes with confidence intervals.

Specifically, the overall OHRQoL impact, as directly reported by the children’s self-reports (21.76%), was more than twice that of the parents’ proxy reports (10.33%). This discrepancy in assessment may not only further confirm that MIH has a much more profound impact on the child’s well-being than parents or caregivers perceive, but it may also consistently reverberate across each quality-of-life domain, revealing two different perspectives on the child’s condition. Rather than indicating that one perspective is more accurate than the other, this discrepancy may reflect differences in how children and parents/caregivers perceive and report the consequences of MIH. These findings are consistent with those reported in several studies, which demonstrate a marked lack of agreement between proxy and children’s reports when assessing the impact of enamel defects, potentially suggesting that the two generational groups experience and interpret the condition through two parallel but non-communicating subjective realities [42,43,44]. Indeed, parents’ attitudes appear to be guided by the visible chromatic and structural impact of the lesion, which is reflected in variations in their willingness to seek a dental consultation [42,43].

Regarding the domain-specific impact on OHRQoL, consistent with the condition’s biological basis, the oral domain emerged as the most affected area in children’s self-reports, with a currently reported percentage impact index of 29.72%.

This trend could be explained by the intrinsic architecture of enamel affected by MIH, which is characterized by reduced mineral density, high macromolecular porosity, and frequent pathological exposure of the underlying dentinal tubules, resulting in chronic and severe thermal, chemical, and tactile hypersensitivity [1,9,10]. Basic daily activities for children, such as drinking cold water, eating sugary or hot foods, and even routine oral hygiene practices like brushing their teeth, may therefore become actual pain triggers. In the most severe cases, the structural fragility of the enamel leads to spontaneous post-eruptive breakdown (PEB), which, by exposing the underlying dentin, paves the way for a potential rapid progression of carious lesions and constant subclinical chronic pulp inflammation [5,6,10,25,45]; this unstable symptomatic scenario may result in deep, persistent pain that may drastically limit the young patient’s ability to carry out normal daily activities and therefore potentially interfere with quality of life.

The oral domain, which comprises oral symptoms and physical pain, has been proven to likely be the only domain to receive partial and concrete recognition from parents, ranking as the value with the highest percentage impact as presently reported by caregivers (13.86%), although it remained underestimated by more than 15 percentage points compared to the child’s actual debilitating experience (29.72%).

This partial convergence may plausibly reflect the greater visibility of physical symptoms compared with psychological or social consequences. Previous studies have reported that pain-related manifestations and changes in daily behavior may be more readily recognized by caregivers [42,43,46].

This difference may also be considered in light of previous evidence showing that parents exhibit greater concern and a stronger sense of urgency when faced with clinically destructive lesions, particularly when associated with visible tooth structure loss and acute pain [42]. Such findings may provide a possible context for interpreting the lower proxy-reported impact observed in the present review, although this specific mechanism was not directly evaluated.

Indeed, parents may show greater concern and a stronger inclination to seek consultation for yellow-brown hypomineralized opacities than for white hypomineralized areas, which, lacking a stark color contrast or an apparent threat of imminent structural collapse, tend to be overlooked or considered of secondary importance by adults [42].

To potentially explain the discrepancy relative to other OHRQoL domains, it is plausible that parents may not be sufficiently informed about the condition’s nature and complexity. Indeed, it has been reported that parents declared having no knowledge of MIH, and about half of them reported that the dentist did not adequately explain either the etiology or the long-term treatment needs [43].

Due to this significant lack of information, parents typically direct their attention and concerns solely toward the immediate management of pain or the potential and imminent consequences of carious lesions and the loss of tooth structure, issues about which they express considerable concern [43,47]. Indeed, parents tend to interpret MIH through a symptom management and clinical framework with a long-term perspective, potentially mainly focusing on the risk of caries or tooth loss, but remain partially unaware of the immediate emotional distress that their child is experiencing.

In the present umbrella review, this discrepancy was particularly evident between the psychological domain (22.42% in children vs. 10.72% in parents) and the social domain (13.26% in children vs. 5.12% in parents).

It follows that, in this context, enhancing the oral health literacy of parents and caregivers is a fundamental component for the prevention and management of pediatric oral health [48,49,50]. Low levels of health literacy among caregivers may result in a deterioration of children’s oral health and a reduced ability to understand and manage their therapeutic needs [48,49,50]. In MIH, a lack of adequate knowledge may lead parents to misinterpret the clinical manifestations and underestimate the condition, both in the primary and permanent dentition. While concerns about permanent teeth primarily may stem from the destructive loss of tooth structure or conspicuous dark discoloration, primary teeth may often be neglected due to the mistaken belief that they have only minor clinical and esthetic importance because of their eventual exfoliation [42,51,52].

However, although this umbrella review evaluated subjects across developmental stages ranging primarily from early mixed dentition to late mixed dentition and permanent dentition, a substantial and essential factor to consider is that a growing body of evidence supports a close biological and predictive correlation between enamel defects in these two stages of dentition, potentially highlighting an epidemiological and phenotypic association between the presence of hypomineralized second primary molars (HSPMs) and the subsequent onset of MIH in the permanent sectors [1,3,9,53]. This biological link underscores the crucial importance of HSPMs not only as a potential early diagnostic predictor but also as a target for timely preventive interventions; consequently, promoting parents’ oral health literacy regarding primary teeth has the potential to make it possible to detect structural defects years before the permanent teeth erupt, thereby reducing the risk of hypersensitivity, caries, and future tooth loss.

It has been reported that pediatric patients with MIH exhibited high levels of concern and profound distress regarding their smile, as they were fully aware that their peers noticed the altered color of their anterior teeth [43,44]. Nevertheless, proxy reports indicated that most parents believed that this esthetic alteration had no impact on their child’s perceived self-confidence or daily social interactions [43,44]. Similarly, in the present umbrella review, the psychological and social domains assessed through children’s self-reports showed greater impact than those estimated by parents, potentially highlighting that the smile serves as a relevant social mirror for personal perception, self-esteem, and overall well-being in interactions with other children.

In addition, it has been widely reported that peers formed immediate, dismissive judgments about smiles affected by MIH, unfairly associating them with reduced social competence, lower leadership potential, and poorer academic success, leading even to bullying and teasing at school [54,55]. To cope with this relational isolation, the child frequently adopted avoidance-based strategies, deliberately stopping smiling freely and even covering their mouth while speaking or laughing [44]. Parents, however, being completely unaware of these extra-domestic dynamics, almost completely failed to perceive the social distress, potentially mistaking their child’s silence or protective avoidance for a calm, clinical acceptance of the condition [44].

Consequently, based on these findings, it may be important to recognize that MIH should not be managed exclusively from a clinical perspective focused solely on the absence or resolution of physical and/or functional oral symptoms. Rather, the therapeutic approach should also consider the oral and functional domains of OHRQoL, as well as the profound impact that this condition may have on psychological, emotional, and relational aspects, potentially protecting and restoring the young patient’s overall well-being and OHRQoL domains.

**Figure 5:**
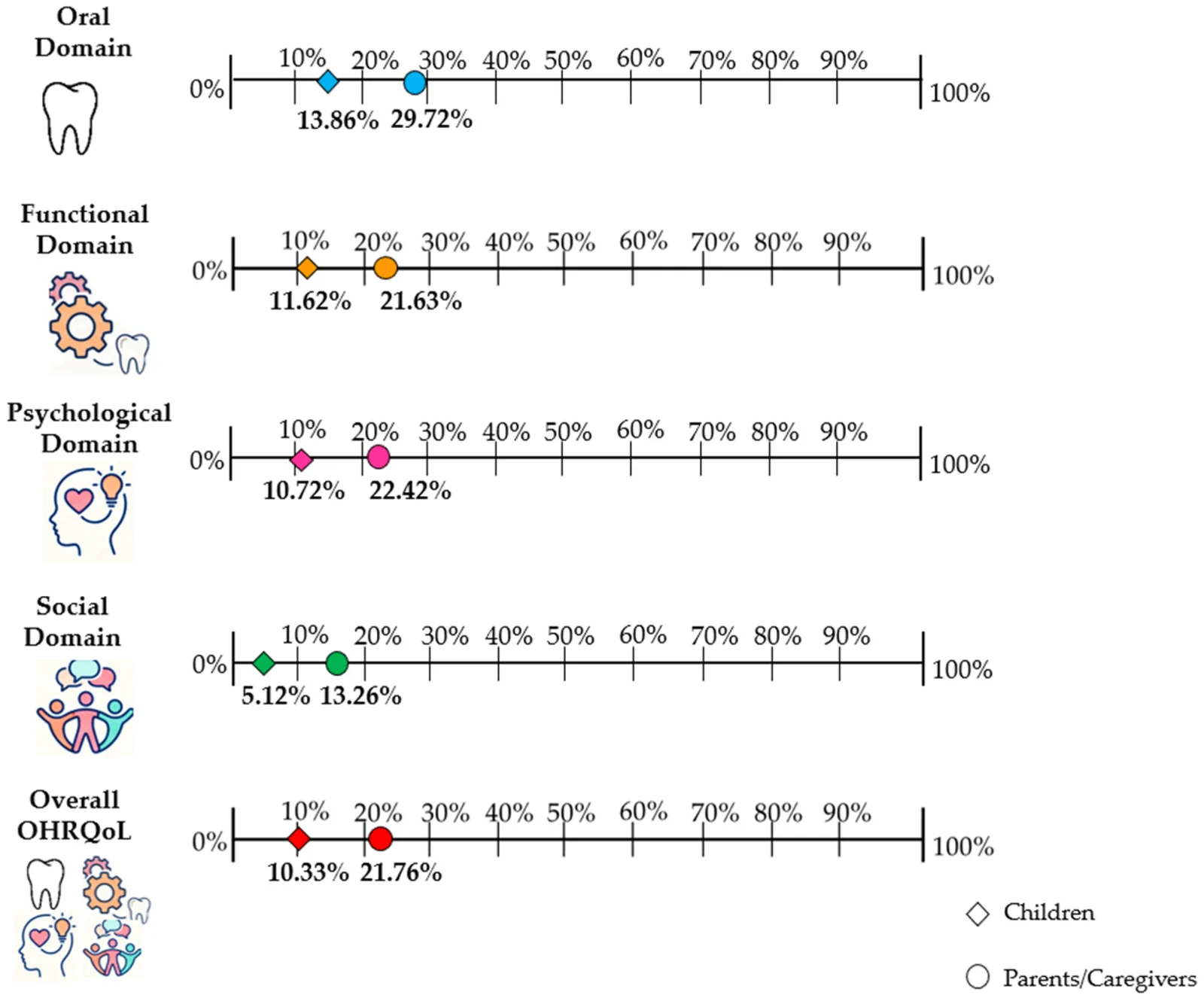
Overall and domain-specific OHRQoL impacts according to children and parents/caregivers. The color coding corresponds to the evaluated domains: blue indicates the oral domain, orange the functional domain, pink the psychological domain, green the social domain, and red the overall OHRQoL. Diamonds represent children’s assessments, whereas circles represent parents/caregivers’ assessments.

Figure 5 shows the overall and domain-specific OHRQoL impacts according to children and parents/caregivers.

### 4.2. Overall and Domain-Specific OHRQoL: Severity/Extent/Distribution Pattern and Age/Dentition Status Impact

In the present umbrella review, the analysis of OHRQoL by severity level was evaluated in a limited subsample of 494 pediatric subjects aged 8 to 10 years, in the early mixed dentition phase, of whom 180 had a mild form, 88 a moderate form, and 226 a severe form; given this limited sample size and the particularly limited subgroups of participants, particularly in the moderate form, current findings should be interpreted as exploratory.

The overall impact of OHRQoL, expressed as the percentage impact index, in subjects with MIH was 14.18% in mild cases, 11.31% in moderate cases, and 21.20% in severe cases.

Although these findings support the widely reported trend in the literature that greater clinical severity of MIH is associated with more marked impairment in oral health-related quality of life, the present umbrella review suggests that this relationship is likely more complex than a simple linear trend. Indeed, rather than showing a gradual increase in the impact of the condition, especially for moderate forms in which there is no proportional increase in the percentages of impact on OHRQoL, the present findings potentially describe a progressive qualitative transformation of the burden perceived by the child, suggesting that the advancement of MIH may alter not only the overall impairment in OHRQoL, but also the dimensions through which the disease is experienced.

This interpretation appears consistent with the natural history of MIH, since in mild forms, the clinical manifestation is generally characterized by the presence of well-defined opacities, with limited structural involvement and symptoms that are often mild or intermittent. Although these changes may cause esthetic discomfort and sensitivity to thermal or mechanical stimuli, in most cases the tooth retains substantial functional integrity [1,6,19,56]. Conversely, as the disease progresses, reduced enamel mineralization, increased porosity, and greater tissue fragility contribute to post-eruptive enamel collapse, dentin exposure, the onset of persistent hypersensitivity, increased susceptibility to caries, and the need for repeated restorations or progressively more invasive treatments [1,6,19,56]. It is therefore plausible that, as the clinical presentation worsens, the child’s perception of the condition may also change, shifting from a predominance of more relevant esthetic and emotional implications to one in which pain, functional limitations, and difficulties in daily activities become the primary factors responsible for the reduction in quality of life. In this context, one of the most relevant aspects that emerged presently concerns the percentage impact values of moderate cases, which showed an overall slightly lower impact than mild cases (11.31% vs. 14.18%). Although this apparent contradiction should be interpreted with caution, especially given the small size of the moderate subgroup, it may reflect the very heterogeneous nature of this clinical category. Unlike mild and severe forms, which are generally characterized by relatively well-defined phenotypes, moderate forms likely encompass a broad spectrum of intermediate conditions, in which different combinations of enamel opacity, initial dentin involvement, hypersensitivity of varying intensity, and atypical restorations that are still functionally effective may coexist [1,6,19,56]. This heterogeneity could have reduced the observed percentages, contributing at least in part to the potential counterintuitive trend observed in the present umbrella review.

The domain-specific analysis showed that the oral domain was consistently the most impaired across the entire severity spectrum, with values of 19.00% in mild cases, 18.00% in moderate cases, and 32.00% in severe cases. Similarly, the impact in the functional domain ranged from 12.70% and 12.50% observed in mild and moderate forms, respectively, up to 24.50% in severe forms. This trend appeared to reflect condition progression, in which higher protein content and increased enamel porosity lead to decreased tissue mechanical properties, facilitating the transmission of thermal and mechanical stimuli to the dentin and predisposing the patient to the development of hypersensitivity [1,6,19,56]. With the onset of post-eruptive breakdown and dentin exposure, pain may become progressively persistent, interfering with daily activities, and in severe cases, oral and functional domains become the primary determinants of the child’s reduced quality of life, as observed in the present umbrella review [1,6,19,56].

This interpretation should be considered in light of the characteristics of the subsample currently analyzed, which consisted of children aged 8 to 10 years. Previous evidence suggested that younger children may report greater sensitivity associated with MIH than older children, although the mechanisms underlying this difference were not directly evaluated in the present review [6,57]. This difference may partly contribute to the greater impact observed in the oral and functional domains. However, differences across age groups and dentition stages should be interpreted cautiously, particularly given the heterogeneity of the OHRQoL instruments used. Consistently, previous studies have reported a greater OHRQoL impact in children with more severe MIH, with oral symptoms and functional limitations representing potential relevant contributors to perceived burden [58,59,60,61].

Although psychological impairment reached a higher value in severe forms (25.00%), it was already compromised in mild (16.10%) and moderate (13.50%) forms, suggesting that emotional distress may represent a relevant component of the disease from its earliest stages. In the social domain, the highest value was observed in moderate forms (11.25%), followed by mild forms (8.90%), while it decreased in severe forms (3.30%). This trend suggests that psychosocial impairment may not depend exclusively on the biological severity of the lesion but might be influenced by the perceived appearance of the child’s smile at different stages of the disease’s progression.

While not directly assessed in the included systematic reviews, it is plausible that when symptoms are relatively limited, visible anterior opacities may represent an important esthetic and social concern. Previous evidence has highlighted the potential relevance of peer perceptions and esthetic concerns in shaping children’s experiences of MIH, with possible implications for perceived OHRQoL, particularly in the psychological and social domains, through factors such as reduced self-esteem, smile dissatisfaction, and difficulties in social relationships [55,62,63,64,65].

In the present umbrella review, none of the included systematic reviews assessed OHRQoL sorted by the number of teeth affected and/or lesion extension/pattern. However, the lack of evidence regarding the number of teeth affected, the anatomical distribution of lesions, or the involvement of the anterior/posterior regions highlights a notable gap in the current literature and likely suggests that the extent and distribution pattern of MIH may represent potential modifiers of the condition’s impact on quality of life, requiring further investigation in future studies.

Indeed, it may be reasonable to hypothesize that the extent of the condition could affect the child’s perceived OHRQoL. For instance, hypomineralization confined to a single permanent first molar, in the absence of marked hypersensitivity or post-eruptive enamel loss, could have a relatively limited impact, allowing the child to spontaneously adapt their masticatory function without substantial limitations in daily life.

Conversely, simultaneous involvement of all four permanent first molars, especially when associated with persistent symptoms, post-eruptive breakdown, and the need for repeated restorations, could likely result in a considerably greater burden, with repercussions primarily in the oral and functional domains. In such circumstances, symptoms in multiple quadrants could limit bilateral chewing, interfere with food texture choices, make routine oral hygiene practices painful, and compromise essential daily activities, such as eating and performing adequate and regular oral hygiene at home [66,67]. Furthermore, pain and hypersensitivity during toothbrushing may reduce patient compliance with daily oral hygiene practices, impairing effective biofilm control and potentially increasing the risk of plaque accumulation, caries progression, and further deterioration of oral health [58,67,68].

Similarly, the distribution pattern of lesions could also be a relevant factor influencing OHRQoL in pediatric subjects. It is plausible that the predominant involvement of the posterior region primarily exerts a functional impact, whereas the extensive involvement of the permanent incisors, especially when opacities exhibit a marked color contrast with healthy enamel, may be of greater significance for esthetic and psychosocial perceptions. Defects localized in the anterior region are immediately visible when smiling and during social interactions and may amplify feelings of embarrassment, reduced self-esteem, and increased awareness of the child’s dental appearance, with repercussions in the psychological and social domains [14,55]. In fact, the mere presence of MIH is not sufficient to determine an impairment in quality of life; rather, it is the way in which specific clinical characteristics of the condition, such as the involvement of permanent incisors, the extent of the lesions, and their visibility when smiling, may influence the child’s esthetic perception and psychosocial well-being [6,14,69].

Severity, extent, and distribution patterns may not act as independent determinants, but rather represent closely interrelated factors capable of synergistically modulating the overall burden of the disease. For example, a clinically moderate form characterized by the involvement of numerous teeth or by extensive opacities localized in the anterior region could theoretically have a greater impact on quality of life than a biologically more severe form limited to a single posterior molar. Similarly, the simultaneous involvement of multiple symptomatic posterior teeth could compromise masticatory function and daily activities to a greater extent than isolated but asymptomatic anterior defects. From this perspective, a child’s OHRQoL may result from the interaction between clinical severity, the number of teeth involved, the anatomical distribution of the lesions, the presence of symptoms, and the functional or esthetic impact of the affected teeth, rather than from the individual and specific clinical and anatomical characteristics of MIH. Therefore, the lack of studies that have systematically evaluated the role of extent and distribution pattern represents a gap identified by this umbrella review. Future studies should therefore incorporate these variables into analyses of OHRQoL to determine whether they are independent factors contributing to impaired quality of life or whether they act as modifiers of the effect of clinical severity in OHRQoL perceptions among pediatric patients with MIH and their parents/caregivers.

**Figure 6:**
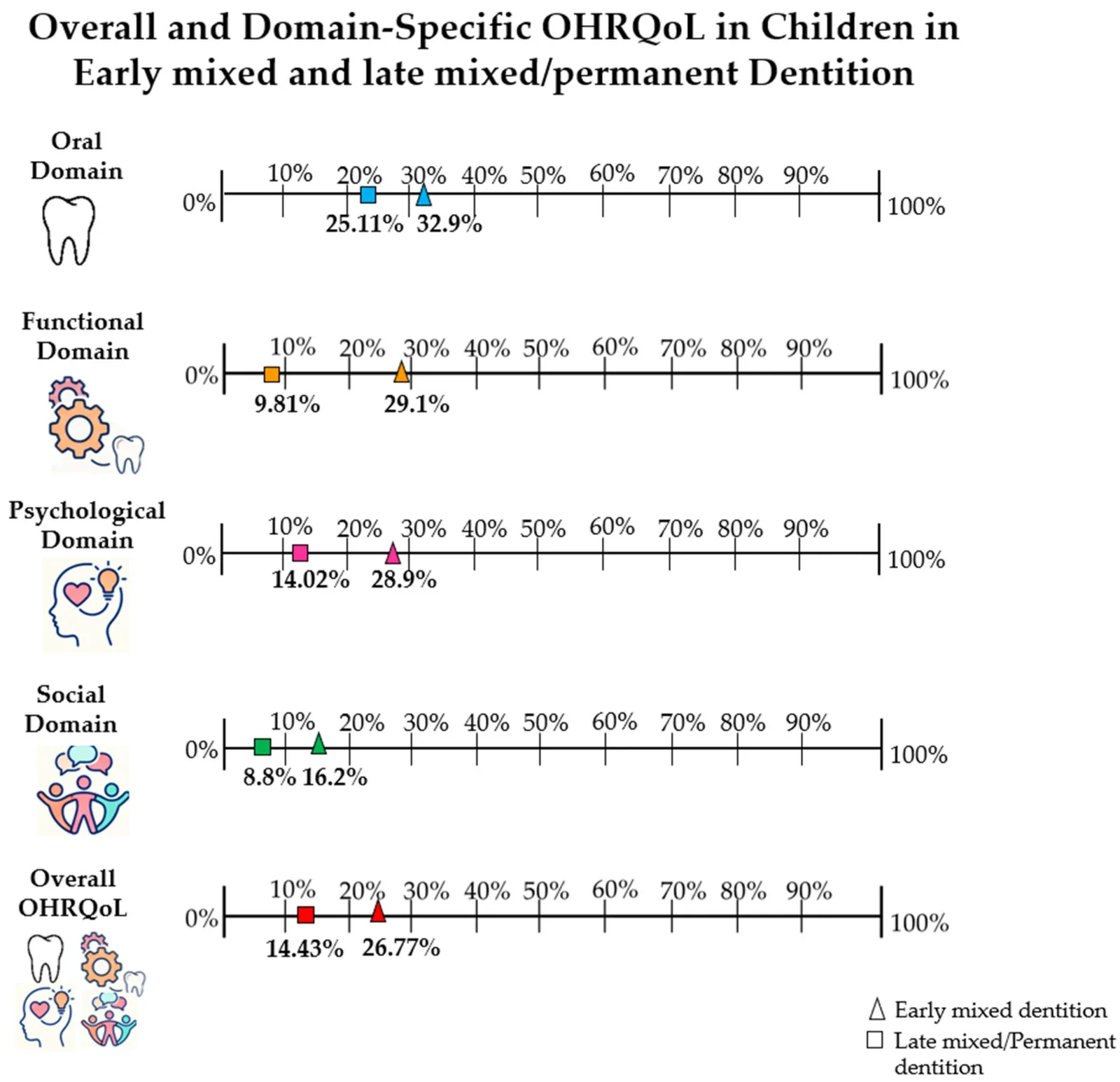
Overall and domain-specific OHRQoL according to dentition status. The color coding corresponds to the evaluated domains: blue indicates the oral domain, orange the functional domain, pink the psychological domain, green the social domain, and red the overall OHRQoL. Triangles represent early mixed dentition, whereas squares represent late mixed/permanent dentition.

The present umbrella review revealed a complex and multifaceted relationship between age and stage of dentition (Figure 6). In fact, interpreting the results may require considering both the stage of dental development at which the child experienced MIH and the conceptual characteristics of the tools used to measure OHRQoL. Rather than suggesting a simple increase or a gradual reduction in impact with age, the data from this umbrella review should be interpreted carefully, as differences between dentition stages may partly reflect differences in the OHRQoL instruments used.

The reported overall OHRQoL impact in children in the early mixed dentition phase was 26.77%, while in the late mixed/permanent dentition phase it was 14.43%.

However, by analyzing the types of questionnaires used in the study population from the Child Perceptions Questionnaire (CPQ) family, children aged 8–10 years, corresponding to the early mixed dentition phase, showed an overall impact on quality of life that was nearly double that of subjects assessed using the CPQ 11–14 (26.77% vs. 14.43%). This difference was observed across all domains investigated and was particularly evident in the oral domain (32.9% vs. 21.0%), the functional domain (29.1% vs. 9.2%), the psychological domain (28.9% vs. 8.6%), and the social domain (16.2% vs. 4.3%). These findings may indicate a greater reported impact among children assessed with the CPQ 8–10; however, this difference cannot be interpreted as evidence of a reduction in OHRQoL impact with age, given the different instruments and age groups considered. This relevant impact observed in early ages was previously reported [70], demonstrating that in children with mixed dentition, the presence of untreated cavities was strongly and independently associated with worse self-rated oral health, confirming that untreated structural lesions and associated pain constituted the main clinical determinant of a negative perception of subjects’ oral health from an early age in childhood.

However, limited comparison of the values obtained using the CPQ 8–10 and the CPQ 11–14 could lead to incomplete conclusions. Indeed, the apparent reduction in impact observed in older subjects was not confirmed by the other instruments used in the same age group. On the contrary, both the C-OHIP-SF19, used in children and preadolescents in the late mixed dentition stage, and the OHIP-14, applied primarily to subjects in the late mixed/permanent dentition stage, documented higher impact values specifically in the oral and psychosocial domains. In the C-OHIP-SF19, for example, the oral domain reached a percentage impact index of 43.7%, while the socioemotional domain showed an impact of 43.9%. Similarly, in the OHIP-14, physical pain reached 50.5%, psychological distress 42.9%, and social disability 24.1%. Taken together, these findings suggest that the apparent differences across dentition stages should not be interpreted as a simple age-related change in OHRQoL impact, but rather considered in relation to both developmental stage and instrument characteristics.

One possible explanation for this apparent discrepancy may lie in the profound conceptual differences between the assessment tools. Although the CPQ 11–14 was specifically developed for the pediatric population, it includes numerous items related to activities of daily living, such as school attendance, homework, and participation in recreational activities [71,72]. In contrast, instruments such as the C-OHIP-SF19 and the OHIP-14 appear to explore the subjective dimension of distress in greater depth, directly assessing emotions such as tension, embarrassment, frustration, irritability, or relationship difficulties [73,74]. These conceptual differences may contribute to the variability observed across instruments and should therefore be considered when interpreting differences between age and dentition groups.

Previous studies have nevertheless reported differences in OHRQoL according to age and psychosocial characteristics. For example, subjects aged 10–15 years have shown significantly greater overall impact than younger children, with differences across oral, functional, emotional, and social domains [61]. Similarly, Carneiro et al. [75] highlighted the potential influence of self-esteem on OHRQoL during the transition from mixed to permanent dentition. Although these findings provide relevant context, they should be considered supporting evidence rather than evidence of mechanisms underlying the age-related OHRQoL differences observed in the present umbrella review.

Overall, the findings of this umbrella review should be interpreted descriptively rather than as demonstrating a progressive reduction or transformation of MIH-related OHRQoL burden with age. Rather, the observed differences across dentition stages may reflect a combination of developmental stage, clinical characteristics, and, importantly, the conceptual differences among the OHRQoL instruments used.

### 4.3. Overall and Domain-Specific OHRQoL: Dental Treatment Impact

The present umbrella review highlighted that dental treatments in pediatric patients with MIH led to potential improvements in overall and domain-specific OHRQoL. However, the overall percentage impact improvement (Δ) and its distribution across individual domains varied considerably by intervention and the child’s perceived baseline disease burden.

However, in this context, when interpreting the findings of the present umbrella review, rather than assuming that a specific treatment determined a fixed degree of improvement, each outcome should ideally be contextualized against baseline values, taking into account the potential impact of disease severity and patient-perceived symptoms, although directly establishing these specific associations remains unfeasible from the findings of the present umbrella review. Also, the calculated net therapeutic gains reported should be interpreted as descriptive mathematical differences between baseline and post-treatment scores, illustrating relative variations in perceived burden rather than proving that an intervention eliminates a fixed percentage of overall impairment.

Moreover, heterogeneous follow-up intervals across primary studies may limit direct comparability among interventions and prevent definitive conclusions regarding the long-term durability of treatment-related OHRQoL gains; thus, these findings should be interpreted as descriptive evidence of the overall and domain-specific impact of dental interventions on OHRQoL.

Specifically, the preventive and desensitizing strategies investigated—which included fissure and pit sealants (resin-based or glass ionomer), topical desensitizing agents, and Er:YAG laser applications—achieved the greatest overall post-treatment percentage improvement (ΔOHRQoL = 22.14%, with the impact decreasing from a baseline of 28.09% to 5.95%). The therapeutic gain was predominantly concentrated in the oral domain (Δ = 36.46%, from a baseline of 47.16% to 10.70%), with significant secondary benefits in the psychological (Δ = 21.75%) and functional (Δ = 19.50%) domains. Indeed, the porous, protein-rich architecture of hypomineralized enamel may facilitate rapid fluid movement within exposed dentinal tubules, triggering acute neurogenic inflammation and severe spontaneous hypersensitivity; therefore, preventive protocols may directly promote the elimination of the primary peripheral nociceptive input. This treatment could lead to rapid symptom remission and restoration of oral function, as demonstrated by the findings of this umbrella review. Furthermore, long-term evidence indicates that sealants applied with an intermediate adhesive fluid ensure optimal retention rates in mild lesions, representing a potential first line of defense against PEB and the onset of secondary caries [1,76].

Minimally invasive esthetic treatment, such as enamel microabrasion, resin infiltration, teeth whitening, and direct composite bonding, resulted in a significant overall improvement in OHRQoL of Δ = 14.31% (from 37.10% to 22.79%). In contrast to preventive measures, the therapeutic impact was strongly skewed toward the psychological (Δ = 18.65%, from a baseline of 43.85% to 25.20%) and social (Δ = 18.65%, from a baseline of 43.85% to 25.20%) domains, with more modest gains in the oral (Δ = 14.45%) and functional (Δ = 5.50%) domains. Delimited and visible opacities, especially on permanent incisors, may constitute a primary source of esthetic dissatisfaction and social embarrassment in the pediatric population, potentially leading to avoidance behaviors and social isolation [77]. Micro-invasive resin infiltration may alter the refractive index of porous enamel, successfully masking white and yellow-brown opacities [77]. Recent clinical evidence confirms that effective masking of anterior MIH lesions may reduce instances of peer teasing and restore self-esteem and confidence in interpersonal relationships [77]. Consequently, esthetic management of anterior MIH should not be viewed as a mere cosmetic procedure, but as a potential intervention that may help mitigate peer stigma and promote healthy emotional development in pediatric patients.

Direct restorative treatments, such as the removal of caries combined with direct composite resins or glass ionomer restorations, resulted in an overall improvement in OHRQoL of Δ = 16.25% (from a baseline of 25.23% to 8.98%). It is noteworthy that the domain-specific distribution revealed that the most marked benefit occurred in the psychological domain (Δ = 24.14%, from a baseline of 32.97% to 8.83%), closely followed by the oral domain (Δ = 23.66%, from a baseline of 38.33% to 14.67%), while functional (Δ = 10.69%) and social (Δ = 6.50%) improvements were more limited. This pattern suggests that restoring a carious and structurally compromised tooth does more than simply eliminate local physical discomfort; it radically transforms the child’s internal perception of their own oral health. Restoring a tooth previously perceived as being broken, painful, and subject to continuous deterioration may instill a deep sense of psychological reassurance, alleviating the constant fear of sudden fractures or pain during chewing.

The restorative treatment of teeth affected by MIH may present a challenge in pediatric dentistry [78,79], as hypomineralized enamel exhibits a disorganized prismatic structure, a high residual protein content, and reduced microhardness, factors that severely compromise enamel–resin bond strength and predispose restorations to marginal microleakage, marginal discoloration, and early loss [80]. Evidence suggests that cavity preparation involving the removal of porous and brittle enamel until a healthy, hard margin is reached could ensure superior long-term restoration survival, compared to non-invasive or partial-removal approaches [76,80]. Indeed, to maintain OHRQoL improvements over time, addressing potential restorative challenges might be clinically relevant. In this context, optimizing substrate conditioning, exploring bioactive restorative approaches, adopting thorough surface finishing protocols, or considering indirect digital solutions for severely affected molars have been suggested as potential additional strategies that could help reduce restoration failures and minimize potential repeated interventions, which may foster a long-term positive impact on the perceived OHRQoL [78,81,82].

When structural destruction progresses unnoticed due to rapid caries-induced progression or delayed intervention, the lesion may inevitably affect the dentin–pulp complex, making endodontic therapy necessary [1,79,80]. In such cases, contemporary endodontic strategies, including active irrigation and disinfection protocols and instrumentation approaches, may potentially represent supportive approaches to manage symptoms and promote potential sustained improvements in OHRQoL over time [83,84,85].

In the present umbrella review, the combined interventions showed a net percentage improvement in overall OHRQoL (Δ = 12.15%, from a baseline of 21.37% to 9.22%), although they yielded balanced benefits across the oral (Δ = 16.00%), psychological (Δ = 15.45%), functional (Δ = 14.10%), and social (Δ = 3.05%) domains. Combined treatment strategies, encompassing preventive, desensitizing, restorative, endodontic, prosthetic, and surgical procedures, were indicated based on the clinical phenotype and severity, and this would explain the lower percentage of impact currently observed, as it depended on the heterogeneity in the perception of the child’s clinical presentation, as well as on the differing perceived potential impact of the various interventions.

**Figure 7:**
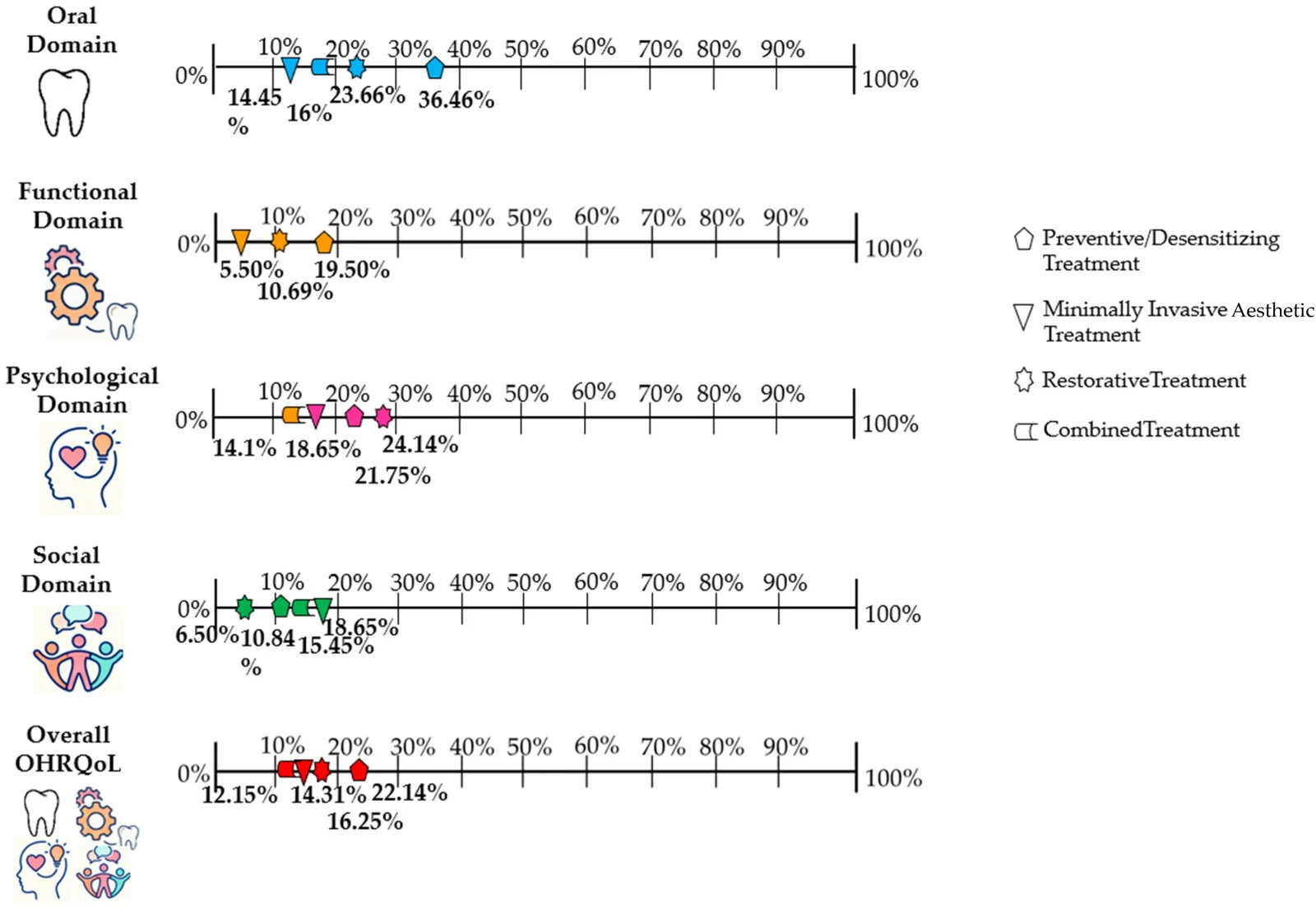
Overall and domain-specific OHRQoL and net therapeutic gain, according to the different dental treatments (preventive/desensitizing; minimally invasive esthetic; restorative; combined). The color coding corresponds to the evaluated domains: blue indicates the oral domain, orange the functional domain, pink the psychological domain, green the social domain, and red the overall OHRQoL. Shapes represent the treatment strategies: pentagons indicate preventive/desensitizing treatment, inverted triangles indicate minimally invasive aesthetic treatment, stars indicate restorative treatment, and c-shapes indicate combined treatment.

Figure 7 depicts the net therapeutic gain (Δ) according to the different treatment strategies.

When severe tissue breakdown precludes tooth restoration, planned extractions, integrated with potential orthodontic space management, may be needed to alleviate pain and functional limitation [76]. However, unmanaged early tooth loss, particularly in anterior esthetic areas, might negatively affect emotional and social well-being due to peer perception [76,86,87]. In this context, temporary space management solutions with esthetic tooth replacement have been suggested as potential options to preserve smile and social confidence during growth, before evaluating fixed solutions upon the completion of skeletal development [76,88,89,90].

However, although a wide range of dental treatments were assessed in the present umbrella review, comprising preventive/desensitizing treatments (pit and fissure sealants, topical desensitizers, Er:YAG laser), minimally invasive esthetic procedures (enamel microabrasion, resin infiltration, bleaching, direct composites), restorative therapies (caries removal and glass hybrid restorations), and combined multi-modal strategies (preventive, esthetic, restorative, endodontic, prosthetic, and surgical procedures), it should be noted that these interventions differed substantially in follow-up periods: in particular, ranging from 3 to 5 months for preventive/desensitizing protocols, 1 month for esthetic approaches, and 6 months for restorative treatments, while remaining undefined for combined interventions. This heterogeneity may limit the generalizability of the findings and the direct comparability among different treatment modalities, indicating that the reported findings should be interpreted as descriptive percentage indices of improvement from baseline rather than definitive conclusions regarding long-term therapeutic durability or sustained OHRQoL stability. These variations should not be interpreted as evidence of clinical efficacy or treatment superiority, as each intervention may address distinct indications; rather, they should be contextualized by both patient-oriented and MIH-related factors. Therefore, considering the present findings as a standardized degree of improvement in OHRQoL would represent an oversimplification; they should serve as descriptive metrics of the treatment impact on OHRQoL by considering baseline vs. post-treatment variations.

Therefore, rather than identifying a single treatment modality that is inherently superior to another, the clinician should guide their decisions by carefully considering the specific dimension of OHRQoL most affected in the individual patient, weighing this subjective perception in close relation to the overall clinical picture—namely, the severity of the lesions, the condition of the dentition, the age of the pediatric patient, the presence and extent of symptoms, and the anatomical distribution pattern of the defects. Further studies are therefore needed to investigate the impact on quality of life in pediatric patients with MIH, taking into account these clinical aspects of the lesions as well as variables related to individual perception.

### 4.4. Strengths, Limitations, and Future Directions

This umbrella review revealed several strengths, along with some limitations that point the way toward future research directions, which should be taken into account when interpreting the current findings.

A relevant strength lies in providing a comprehensive, systematic, and in-depth assessment of the impact of MIH on oral health-related quality of life, analyzing both the overall and specific domains of OHRQoL, from the perspectives of children, parents, and caregivers, while also considering the influence of disease severity, the extent and distribution pattern of lesions, age, stage of dentition, and different dental treatment options. Furthermore, from a methodological standpoint, a normalization calculation was performed to descriptively compare data from questionnaires with inconsistent metric ranges and structures by converting the scores into a descriptive normalized percentage index; however, it should be recognized that these are author-derived descriptive metrics rather than standardized pooled measures.

Nevertheless, this study has some inherent limitations that warrant consideration. First, the relatively limited number of eligible systematic reviews, with six reviews ultimately included, should be acknowledged. Although these reviews collectively provided a broad overview of the available evidence, the number of syntheses meeting the eligibility criteria remained limited, which may have constrained the breadth of the evidence base and the ability to explore specific clinical and methodological subgroups in greater depth.

Moreover, the included systematic reviews showed very high primary study overlap, with a CCA value of 31.43%. This finding was largely expected given the narrow, specific aim of the research topic and the close publication timeframe of the included studies (published between 2021 and 2026), which inevitably led them to draw on the same core body of original evidence. Consequently, this high degree of overlap should be carefully considered when interpreting the overall findings of this umbrella review, as the synthesis relies on a shared pool of primary evidence rather than completely independent study populations.

Furthermore, the methodological quality of the included systematic reviews was variable, representing an additional consideration when interpreting the findings. According to the AMSTAR-2 assessment, none of the included reviews was rated as being of high overall methodological quality; two were classified as moderate quality (33.33%), two as low quality (33.33%), and two as critically low quality (33.33%). This variability in methodological quality may have influenced the consistency of the available evidence and should therefore be considered when interpreting the overall findings of this umbrella review.

In addition, the marked methodological heterogeneity of the primary data and the variability in reporting formats precluded the possibility of conducting a formal quantitative meta-analysis, limiting the synthesis to a descriptive and comparative level. Consequently, the normalized descriptive percentage scores and the 0–100 scale conversion should be interpreted with caution, as they are solely intended as descriptive comparative metrics rather than statistical estimates derived from meta-analytic pooling. Specifically, the overall and domain-specific descriptive impact percentages in children and parents/caregivers should be treated as author-derived descriptive indices constructed to harmonize scales with discordant scoring systems, rather than conventional meta-analytic estimates with confidence intervals.

Furthermore, a descriptive normalized score reflects solely the relative position within the instruments’ theoretical impairment scale (0–100%) and should not be interpreted as a literal or fixed percentage reduction in overall or domain-specific OHRQoL caused by MIH, nor should the net therapeutic gain be considered an absolute or fixed rate of clinical improvement across interventions.

Additionally, calculating the overall OHRQoL impact as the unweighted arithmetic mean across the four individual domains inherently assumes an equal relative weight among oral symptoms, functional limitations, emotional/psychological well-being, and social well-being dimensions. While this approach may methodologically reflect the framework of validated OHRQoL instruments (e.g., CPQ, OHIP) to provide a neutral descriptive summary, it represents a potential methodological simplification. Indeed, pediatric patients and caregivers may assign different subjective priorities to each domain, so specific OHRQoL dimensions could disproportionately influence overall perceived quality of life.

Furthermore, the included evidence showed a limited availability of data concerning the extent of lesions and the anatomical distribution pattern of defects; additionally, the assessment of impact based on severity was based on a numerically small sample subgroup, particularly for moderate forms, and many reviews aggregated age groups that were too broad to allow for a detailed analysis based on the stage of dentition.

Finally, potential language and indexing biases should also be considered. The search strategy was restricted to studies published in English and indexed in the selected electronic databases, which may have excluded relevant non-English research or studies present in other databases.

Building upon these findings, future research should aim to provide a more comprehensive understanding of the multidimensional impact of MIH on OHRQoL by integrating clinical, demographic, developmental, and patient-centered factors, while also focusing on identifying the determinants of this burden and how they interact throughout childhood. Rather than considering MIH as the sole determinant of OHRQoL impairment, future studies should clarify how disease severity, lesion extent and distribution pattern, age, dentition stage, symptoms, and the child’s own perception interact to shape both overall OHRQoL impairment and the relative contribution of its individual domains. Furthermore, investigating the agreement and discrepancies between child- and parent-reported outcomes may help explain part of the heterogeneity currently observed across studies, improve the interpretation of patient-reported outcomes, and facilitate more accurate risk stratification and individualized therapeutic decision-making. Finally, future prospective studies should determine whether different treatment strategies exert domain-specific effects on OHRQoL according to the patient’s clinical phenotype, thereby supporting the development of personalized, precision-based, patient-centered management approaches for MIH.

## 5. Conclusions

Molar incisor hypomineralization has a considerable, negative, and multidimensional potential impact on oral health-related quality of life in the pediatric population. Across the synthesized evidence, this impairment may be particularly noticeable in the oral and symptomatic domains, followed by the psychological, functional, and social domains.

Children consistently appeared to perceive a greater impact of the condition than their parents/caregivers estimate, potentially highlighting a relevant underestimation. Indeed, parents and caregivers might focus their attention primarily on oral and functional symptoms, which could lead to pronounced discrepancies compared with children’s subjective perceptions in the psychological and social domains.

Children in early mixed dentition stage reported a higher descriptive percentage impact than those in the late mixed or permanent dentition stage; however, this difference should not be viewed as an established age-related reduction, as it may largely reflect the conceptual diversity and scoring properties of the specific instruments employed. Similarly, preliminary observations from an exploratory subsample (*n* = 494) suggest that clinical severity might further compromise OHRQoL, particularly in the oral and functional domains, though this tendency should not be assumed as a definitive relationship due to limited subgroup data. In addition, while dental interventions showed descriptive percentage improvements across both overall and domain-specific OHRQoL, these findings derive from a single systematic review evaluating a limited sample (*n* = 491). Consequently, heterogeneity in interventional protocols and variable follow-up periods preclude drawing definitive conclusions regarding the impact or long-term durability of treatment-related OHRQoL gains, suggesting that these findings should be considered predominantly descriptive and exploratory.

Finally, the overall interpretation of the available evidence should take into account notable methodological limitations. None of the six included systematic reviews was rated as high quality according to AMSTAR-2 (two were moderate, two low, and two critically low), accompanied by a very high primary study overlap (CCA = 31.43%) and potential risk of reporting biases. Therefore, while these findings provide valuable initial insights into the pediatric burden of MIH, future studies with standardized outcome measures and extended follow-up intervals would be beneficial to further elucidate the impact of MIH on OHRQoL and support clinical decision-making.

## Figures and Tables

**Table 1:** Domain-specific OHRQoL mean values and impact percentages, sorted by scales, overall domain child impact, overall domain parent-reported impact, and overall domain impact.

	Oral Domain		Functional Domain	Psychological Domain			Social Domain		
	Oral Symptoms	Physical Pain	Functional Limitation	Emotional Well-Being	Psychological Discomfort	Psychological Disability	Social Well-Being	Social Disability	Handicap
CPQ 8–10	6.58 (*n* = 1890); 32.9% [35,38,40]	NA	5.82 (*n* = 1890); 29.1% [35,38,40]	5.78 (*n* = 1890); 28.9% [35,38,40]	NA	NA	6.48 (*n* = 1890); 16.2% [35,38,40]	NA	NA
CPQ 11–14	5.03 (*n* = 1100); 21.0% [37,38,40]	NA	3.30 (*n* = 1100); 9.2% [37,38,40]	3.10 (*n* = 1100); 8.6% [37,38,40]	NA	NA	2.26 (*n* = 1100); 4.3% [37,38,40]	NA	NA
C-OHIP SF19	11.26 (*n* = 93); 43.7% [38]	NA	13.28 (*n* = 93); 17.0% [38]	22.46 (*n* = 93); 43.9% [38]	NA	NA	22.46 (*n* = 93); 43.9% [38]	NA	NA
OHIP-14	NA	4.04 (*n* = 110); 50.5% [38]	NA	NA	3.43 (*n* = 110); 42.9% [38]	1.96 (*n* = 110); 24.5% [38]	NA	1.93 (*n* = 110); 24.1% [38]	1.25 (*n* = 110); 15.6% [38]
Overall domain-specific child impact (%)	29.72%		21.63%	22.42%			13.26%		
Overall child impact (%)	21.76%								
P-CPQ	3.33 (*n* = 835); 13.86% [37,40]	NA	3.72 (*n* = 835); 11.62% [37,40]	3.00 (*n* = 726); 10.72% [37,40]	NA	NA	2.05 (*n* = 726); 5.12% [37,40]	NA	NA
Overall domain parent impact (%)	13.86%		11.62%	10.72%			5.12%		
Overall parent impact (%)	10.33%								
Overall domain impact (%)	26.43%		19.50%	20.25%			11.75%		

Abbreviations: CPQ 8–10, “Child Perceptions Questionnaire for Children Aged 8–10 Years”; CPQ 11–14, “Child Perceptions Questionnaire for Adolescents Aged 11–14 Years”; C-OHIP SF19, “Child Oral Health Impact Profile—Short Form 19”; OHIP-14, “Oral Health Impact Profile—14-item Version”; %, “percentage”; NA, “not applicable”.

**Table 2:** Domain-specific and overall impact on OHRQoL, sorted by dentition status across the various scales, along with the corresponding mean scores and impact percentage.

Dentition Status (Age Range)	OHRQoL Scale/Tool	Oral Domain		Functional Domain	Psychological Domain			Social Domain		
		Oral Symptoms	Physical Pain	Functional Limitation	Emotional Well-Being	Psychological Discomfort	Psychological Disability	Social Well-Being	Social Disability	Handicap
Early mixed dentition (8–10 years (*n* = 1890) [35,38,40]	CPQ 8–10	6.58 (*n* = 1890); 32.9%	NA	5.82 (*n* = 1890); 29.1%	5.78 (*n* = 1890); 28.9%	NA	NA	6.48 (*n* = 1890); 16.2%	NA	NA
	Overall domain-specific child impact (%)	32.9%		29.1%	28.9%			16.2%		
	Overall child impact (%)	26.77%								
Late mixed/ permanent dentition (11–14 years) (*n* = 1303) [37,38,40]	CPQ 11–14	5.03 (*n* = 1100); 21.0%	NA	3.30 (*n* = 1100); 9.2%	3.10 (*n* = 1100); 8.6%	NA	NA	2.26 (*n* = 1100); 4.3%	NA	NA
	C-OHIP SF-19	11.26 (*n* = 93); 43.7%	NA	13.28 (*n* = 93); 17.0%	22.46 (*n* = 93); 43.9%	NA	NA	22.46 (*n* = 93); 43.9%	NA	NA
	OHIP-14	NA	4.04 (*n* = 110); 50.5%	NA	NA	3.43 (*n* = 110); 42.9%	1.96 (*n* = 110); 24.5%	NA	1.93 (*n* = 110); 24.1%	1.25 (*n* = 110); 15.6%
	Overall domain-specific child impact (%)	25.11%		9.81%	14.02%			8.80%		
	Overall child impact (%)	14.43%								

Abbreviation: CPQ 8–10, “Child Perceptions Questionnaire for Children Aged 8–10 Years”; CPQ 11–14, “Child Perceptions Questionnaire for Adolescents Aged 11–14 Years”; C-OHIP SF-19, “Child Oral Health Impact Profile—Short Form 19”; OHIP-14, “Oral Health Impact Profile—14-item Version”; %, “percentage”; NA, “not applicable”.

**Table 3:** Dental treatment impact on domain-specific OHRQoL pre-treatment and post-treatment changes and improvement, expressed as percentages.

Dental Treatment	Follow-Up	Percentage Impact Index	Oral Domain	Functional Domain	Psychological Domain	Social Domain	Domain with Greatest Improvement	Overall OHRQoL
Preventive/Desensitizing Treatment Pit and fissure sealants (resin-based and glass ionomer), Er:YAG laser + topical desensitizer applications (*n* = 148) [38]	3–5 months	Pre-treatment	47.16%	22.50%	27.35%	15.35%	Oral domain	28.09%
		Post-treatment	10.70%	3.00%	5.60%	4.51%		5.95%
		Δ	36.46%	19.50%	21.75%	10.84%		22.14%
Minimally Invasive Esthetic Treatment Enamel microabrasion, resin infiltration, tooth whitening, direct composite restorations (*n* = 93) [38]	1 month	Pre-treatment	43.70%	17.00%	43.85%	43.85%	Psychological/Social domain	37.10%
		Post-treatment	29.25%	11.50%	25.20%	25.20%		22.79%
		Δ	14.45%	5.50%	18.65%	18.65%		14.31%
Restorative Treatment Caries removal, glass hybrid restorations (*n* = 40) [38]	6 months	Pre-treatment	38.33%	20.00%	32.97%	9.62%	Psychological domain	25.23%
		Post-treatment	14.67%	9.31%	8.83%	3.12%		8.98%
		Δ	23.66%	10.69%	24.14%	6.50%		16.25%
Combined Treatment Preventive, minimally invasive esthetic, restorative, endodontic, prosthodontic and extraction procedures according to MIH severity (*n* = 210) [38]	N/d	Pre-treatment	31.80%	24.50%	25.30%	3.88%	Oral domain	21.37%
		Post-treatment	15.80%	10.40%	9.85%	0.83%		9.22%
		Δ	16.00%	14.10%	15.45%	3.05%		12.15%

Abbreviation: %, “percentage”; N/d, “not defined”.

## Data Availability

The datasets generated and analyzed for the present umbrella review, including the data extraction and AMSTAR-2 quality assessments, are available as Supplementary Materials associated with this article.

## Supplementary Materials

The following supporting information can be downloaded at https://www.mdpi.com/article/10.3390/children13091235/s1: File S1: PRISMA checklist; File S2: Data extraction; File S3: Data extracted on OHRQoL domains sorted by scales, MIH severity and age/dentition status; File S4: quality assessment. References [30,35,36,37,38,39,40] are cited in the Supplementary Materials.
