# Supplementary material for: Oral Health-Related Quality of Life in Pediatric Subjects with Molar Incisor Hypomineralization: An Umbrella Review of Domain-Specific Impact and Treatment-Related Changes
Source: Children (Basel). 2026 Sep 12;13(9):1235. doi: 10.3390/children13091235 (PMC13605172; doi:10.3390/children13091235)
Supplement: Supplementary file 1 [file children-13-01235-s001.zip › Supplementary File S2.pdf]

Supplementary File S2: Data Extraction

Table S2. Data extracted from the included studies: Systematic review (First Author, Year, Journal, Reference, Study design, Meta-analysis, Quality); Population (Sample size, Mean age/age range, Gender ratio, Country, Dentition status); Exposure (MIH Classification system, Prevalence, MIH severity, Extent/Distribution pattern, Treatment (if any); Outcomes (OHRQoL classification; Respondents; Overall OHRQoL mean values)

**Systematic Review**<br>First Author<br>Year<br>Journal<br>Reference<br>Study design<br>Meta-analysis<br>Quality	**Population**<br>Sample size<br>Mean age/age range<br>Gender ratio<br>Country<br>Dentition status	**Exposure**<br>MIH Classification system<br>Prevalence<br>MIH Severity<br>Extent/ Distribution pattern<br>Treatment (if any)	**Outcomes(s)**<br>OHRQoL classification<br>Respondents (Child/Parents/Caregiver)<br>Overall OHRQoL mean values
Amrollahi, N. et al.<br>2023<br>*J Evid Base Dent Pract*<br>[35]<br>Cross-sectional (*n*=9); case control (*n*=3); non-randomized clinical trial (*n*=1)<br>Meta-analysis (*n*=9)<br>Low Quality	6504<br>MD/ 6-16 yo (*n*=6504)<br>3825M/2679 F<br>MD<br>Iran (*n*=2507); Germany (*n*=1281); Brazil (*n*=1074); Nigeria (*n*=853); Mexico (*n*=411); Belgium (*n*=290); Columbia (*n*=88)<br>Early mixed dentition (*n*=1508)	EAPD 2003 (*n*=1454); Ghanim 2017 (*n*=54)<br>1508/6504<br>Mild (*n*= 534/1454); Severe (*n*=513/1454); Moderate/severe (*n*=166)<br>MD<br>MD<br>No treatment	CPQ 8-10 (*n*=1508)<br>Child (*n*=1508)<br>Total CPQ 8-10 mean: 15.89 (*n*=1050)
Awwad, A. et al.<br>2023<br>*Acta Stomatol Croat.*<br>[36]<br>Cross sectional (*n*=10); Case-control (*n*=1)<br>Meta-analysis (*n*=11)<br>Critically Low Quality	5017<br>MD/6-16 yo (*n*=5017)<br>MD<br>Brazil (*n*=2676); Germany (*n*=1033); Nigeria (*n*=853); Mexico (*n*=411); Australia (*n*=44)<br>N/d mixed/permanent dentition (*n*=1174)	EAPD (*n*=1062); MIH-TIN (*n*=94); DDE Index (*n*=18)<br>1174/5017<br>Severe (*n*=529)<br>No treatment	CPQ 8-10 (*n*=467); CPQ 11-14 (*n*=177); C-OHIP G-19 (*n*=217); ECHOIS (*n*=124); C-OIDP (*n*=25)<br>Child (*n*=1010)<br>Total OHRQoL values were not reported. The pooled analysis showed that MIH had a negative impact on OHRQoL, but not statistically significant (OR=1.72, 95% Cl:0.99-2.98). However, moderate and severe forms had a significant negative impact on OHRQoL (OR=3.43, 95% Cl:1.69-6.98; P=0.0007)
Hashemi, S. et al.<br>2025<br>*Int J Dent.*<br>[37]<br>Cross-sectional (*n*=9); case control (*n*=1); non-randomized clinical trial (*n*=1)	5861<br>MD/6-16 yo (*n*=5861)<br>3077M/2102F<br>Iran (*n*=2507); Brazil (*n*=1403); Nigeria (*n*=853); Australia (*n*=638); Germany (*n*=317); United Kingdom (*n*=103); Turkey (*n*=40)	EAPD 2003 (*n*=867); DDE Index (*n*=399); Ghanim 2017 (*n*=90)<br>1356/5861<br>Mild (*n*=786/1356); Severe (*n*=444/1356)<br>MD<br>MD<br>No treatment	CPQ 8-10, C-OHIP SF19, COHIP-SF 19, Child OIDP (*n*= N/d); CPQ 11-14 (*n*=697); P-CPQ (*n*=672)<br>Child; Parents<br>Total CPQ 11-14 mean: 13.56 (*n*=697)<br>Total P-CPQ mean: 9.86 (*n*=672)

Meta-analysis (*n*=6)<br>Low Quality	Late mixed/permanent dentition<br>(*n*=1356)		
Hoogeveen, R.P.P. et al.<br>2026<br>Eur Arch Paediatr Dent.<br>[38]<br>Non-randomized clinical trial (*n*=3); case-control (*n*=1); RCT (*n*=1)<br>No meta-analysis<br>Moderate Quality	491<br>9.78 yo/6-16 yo (*n*=491)<br>233M/258F<br>Germany (*n*=210); China (*n*=110); UK(*n*=93); Turkey (*n*=40) Austria (*n*=38);<br>Early mixed dentition (*n*=248); late mixed/permanent dentition (*n*=138)	EAPD 2003 (*n*=491)<br>491/491<br>N/d<br>MD<br>preventive/desensitizing treatment (*n*=148); minimally invasive aesthetic treatment (*n*=93); restorative treatment (*n*=40); combined treatment strategies (*n*=210).	CPQ 8-10 (*n*=143); CPQ 11-14 (*n*=40); C-OHIP SF19 (*n*=93); OHIP 14 (*n*=110)<br>Child (*n*=491)<br>Total CPQ 8-10 mean pre-treatment: 17.03 ±9.66 (*n*=143)<br>Total CPQ 11-14 mean pre-treatment: 33.27± 16.46 (*n*=40)<br>Total C-OHIP mean pre-treatment 47 ±9.29 (*n*=93)<br>Total OHIP mean pre-treatment: 17.61± 4.22 (*n*=110)
Jalevic, B. et al.<br>2021<br>*Eur Arch Paediatr Dent.*<br>[39]<br>Case-control (*n*=1); Cross-sectional (*n*=7)<br>No meta-analysis<br>Moderate Quality	3565<br>MD/6-16 yo (*n*=3565)<br>MD<br>Brazil (*n*=1575); Nigeria (*n*=853); Australia (*n*=638); Mexico (*n*=411); Columbia (*n*=88)<br>N/d mixed/permanent dentition (*n*=3565, of which *n*=831 with MIH)	EAPD 2003 (*n*=695); DDE Index (*n*=136)<br>831/3565<br>Mild (*n*=388); Moderate/Severe (*n*=283)<br>MD<br>MD<br>No treatment	CPQ 8-10 (*n*=451); CPQ 11-14 (*n*=18); Child OIDP (*n*=25); P-CPQ (*n*=362); CPQ 8-10 and CPQ 11-14 (*n*= N/d)<br>Child (*n*=494); Parents (*n*=362)<br>Total OHRQoL values were not reported. The OHRQoL outcomes were narratively analyzed. MIH showed a negative impact on OHRQoL, with a higher impact in severe cases.
Jawdekar, A.M. et al.<br>2023<br>*J Indian Soc Pedod Prev Dent.*<br>[40]<br>Observational study (*n*=6);<br>Meta-analysis (*n*=6)<br>Critically Low Quality	2966<br>6-18 yo (*n*=2966)<br>Brazil (*n*=1575); Nigeria (*n*=853); Mexico (*n*=411); Columbia (*n*=88); Austria (*n*=39)<br>Early mixed dentition (*n*=592); late mixed/permanent dentition (*n*=253);<br>N/d mixed/permanent dentition (*n*=163)	EAPD (*n*=826); DDE Index (*n*=132); Ghanim (*n*=50)<br>1008/2966<br>Mild (*n*=683/1008): Moderate (*n*=193/1008); Severe (*n*=116/1008); N/d (*n*=16)<br>MD<br>MD<br>No treatment	CPQ 8-10 (*n*=592); CPQ 11-14 (*n*=109); P-CPQ (*n*=163)<br>Child; Parents<br>Total CPQ 8-10 mean: 27.44±20.32 (*n*=592)<br>Total CPQ 11-14 mean: 11.92 ±7.98 (*n*=109)<br>Total P-CPQ mean: 22.18 ±15.31 (*n*=163)

Abbreviations: RCT, "Randomized Controlled Trial"; MD, "Missing Data"; yo, "years old"; M, "Male"; F, "Female"; N/d, "Not defined"; EAPD, "European Academy of Paediatric Dentistry"; MIH, "Molar Incisor Hypomineralization"; MIH-TIN, "Molar Incisor Hypomineralization-Treatment Need Index"; DDE Index, "Developmental Defects of Enamel Index"; OHRQoL, "Oral Health-Related Quality of Life"; CPQ 8–10, "Child Perceptions Questionnaire for Children Aged 8–10 Years"; CPQ 11–14, "Child Perceptions Questionnaire for Adolescents Aged 11–14 Years"; C-OHIP G-19, "Child Oral Health Impact Profile – German Short Form 19"; ECHOIS, "Early Childhood Oral Health Impact Scale"; C-OIDP, "Child-Oral Impacts on Daily Performances"; OR, "Odds Ratio"; CI, "Confidence Interval"; C-OHIP SF19, "Child Oral Health Impact Profile – Short Form 19"; P-CPQ, "Parental-Caregiver Perception Questionnaire"; OHIP 14, "Oral Health Impact Profile – 14-item Version".
