# Supplementary material for: Oral Health-Related Quality of Life in Pediatric Subjects with Molar Incisor Hypomineralization: An Umbrella Review of Domain-Specific Impact and Treatment-Related Changes
Source: Children (Basel). 2026 Sep 12;13(9):1235. doi: 10.3390/children13091235 (PMC13605172; doi:10.3390/children13091235)
Supplement: Supplementary file 1 [file children-13-01235-s001.zip › Supplementary File S3.pdf]

Supplementary File S3: Data extracted on OHRQoL domains sorted by Scales, MIH Severity and Age/Dentition status.

Table S3. OHRQoL domains sorted by Scale/Tool; age and dentition status; MIH severity, mean domain score and impact percentage.

OHRQoL Scale/Tool	Mean age/Age range/ dentition status	MIH severity	Domains	Theoretical domains' range	Mean domain score	Impact percentage of domain
**CPQ 8-10**	8.77 (*n*=248)/6-10 yo (*n*=1890) (early)Mixed dentition (*n*=1890) [35,38,40]	N/d	Oral symptom	0-20	6.58 (*n*=1890)	32.9%
			Functional Limitation	0-20	5.82 (*n*=1890)	29.1%
			Emotional well-being	0-20	5.78 (*n*=1890)	28.9%
			Social well-being	0-40	6.48 (*n*=1890)	16.2%
	8-10 yo (*n*=494) Early mixed dentition [35]	Mild (*n*= 180) [35]	Oral symptom	0-20	3.80 (*n*=180)	19.0%
			Functional Limitation	0-20	2.53 (*n*=180)	12.7%
			Emotional well-being	0-20	3.22 (*n*=180)	16.1%
			Social well-being	0-40	3.56 (*n*=180)	8.9%
		Moderate (*n*=88) [35]	Oral symptom	0-20	3.60 (*n*=88)	18.0%
			Functional Limitation	0-20	2.50 (*n*=88)	12.5%
			Emotional well-being	0-20	2.70 (*n*=88)	13.5%
			Social well-being	0-40	0.50 (*n*=88)	1.25%
		Severe (*n*=226) [35]	Oral symptoms	0-20	6.40 (*n*=226)	32.0%
			Functional Limitation	0-20	4.90 (*n*=226)	24.5%
			Emotional well-being	0-20	5.00 (*n*=226)	25.0%
			Social well-being	0-40	1.30 (*n*=226)	3.3%
**CPQ 11-14**	10.71(*n*=150)/11-14 yo (*n*=1100) Late mixed/ permanent dentition (*n*=1100) [37-38,40]	N/d	Oral symptoms	0-24	5.03 (*n*=1100)	21.0%
			Functional Limitation	0-36	3.30 (*n*=1100)	9.2%
			Emotional well-being	0-36	3.10 (*n*=1100)	8.6%
			Social well-being	0-52	2.26 (*n*=1100)	4.3%
**P-CPQ**	6-16 yo (*n*=672) N/d mixed/ permanent dentition [37] **This study was excluded from dentition status analysis**	N/d	Oral symptoms	0-24	2.65 (*n*=672)	11.04%
			Functional Limitation	0-32	3.09 (*n*=672)	9.66%
			Emotional well-being	0-28	1.98 (*n*=563)	7.07%
			Social well-being	0-40	1.71 (*n*=563)	4.27%

	6-12 yo (*n*=163)<br>N/d mixed dentition<br>[40]<br>**This study was excluded from dentition status analysis**	N/d	Oral symptoms	0-24	6.12 (*n*=163)	33.31%
			Functional Limitation	0-32	6.30 (*n*=163)	25.72%
			Emotional well-being	0-28	6.53 (*n*=163)	30.46%
			Social well-being	0-40	3.22 (*n*=163)	10.51%
**C-OHIP-SF19**	10.95±2.54 (*n*=93)<br>Late mixed/permanent dentition<br>[38]	N/d	Oral symptoms	0-20	11.26 (*n*=93)	43.7%
			Functional Limitation	0-16	13.28 (*n*=93)	17.0%
			Emotional well-being	0-40	22.46 (*n*=93)	43.9%
			Social well-being	0-40	22.46 (*n*=93)	43.9%
**OHIP-14**	10.30±1.73/11-14 (*n*=110)<br>Late mixed/permanent<br>[38]	N/d	Physical pain	0-8	4.04 (*n*=110)	50.5%
			Psychological disability	0-8	1.96 (*n*=110)	24.5%
			Social disability	0-8	1.93 (*n*=110)	24.1%
			Handicap	0-8	1.25 (*n*=110)	15.6%
			Psychological discomfort	0-8	3.43 (*n*=110)	42.9%

Abbreviations: OHRQoL, "Oral Health-Related Quality of Life"; CPQ 8–10, "Child Perceptions Questionnaire for Children Aged 8–10 Years"; yo, "Years Old"; N/d, "Not defined "; %, "Percentage"; CPQ 11–14, "Child Perceptions Questionnaire for Adolescents Aged 11–14 Years"; P-CPQ, "Parental-Caregiver Perception Questionnaire"; C-OHIP-SF19, "Child Oral Health Impact Profile – Short Form 19"; OHIP-14, "Oral Health Impact Profile – 14-item Version".
