# Supplementary material for: Oral Health-Related Quality of Life in Pediatric Subjects with Molar Incisor Hypomineralization: An Umbrella Review of Domain-Specific Impact and Treatment-Related Changes
Source: Children (Basel). 2026 Sep 12;13(9):1235. doi: 10.3390/children13091235 (PMC13605172; doi:10.3390/children13091235)
Supplement: Supplementary file 1 [file children-13-01235-s001.zip › Supplementary File S4.pdf]

**Supplementary File S4: Quality assessment**

**Table S4.** Evaluation of the risk of bias of included studies using the AMSTAR-2. First author, year, reference, AMSTAR-2 items to be evaluated, and quality of the study.

| Study | D1 | D2* | D3 | D4* | D5 | D6 | D7* | D8 | D9* | D10 | D11* | D12 | D13* | D14 | D15* | D16 | Quality |
|---|---|---|---|---|---|---|---|---|---|---|---|---|---|---|---|---|---|
| Amrollahi, N. et al. 2023 [35] | Y | Y | Y | Y | Y | Y | PY | Y | Y | N | N | N | Y | Y | Y | Y | Low |
| Awwad, A. et al. 2023 [36] | Y | Y | Y | Y | Y | Y | Y | Y | PY | N | N | N | PY | Y | Y | Y | Critically Low |
| Hashemi, S. et al. 2025 [37] | Y | Y | Y | Y | Y | Y | PY | Y | Y | N | Y | Y | Y | Y | Y | Y | Low |
| Hoogeveen, R.P.P. et al. 2026 [38] | Y | Y | Y | Y | Y | Y | Y | Y | Y | N | NV | NV | Y | Y | NV | Y | Moderate |
| Jalevic, B. et al. 2021 [39] | Y | Y | Y | Y | Y | Y | PY | Y | Y | N | NV | NV | Y | Y | NV | Y | Moderate |
| Jawdekar, A.M. et al. 2023 [40] | Y | Y | Y | Y | Y | Y | PY | Y | PY | N | N | N | Y | Y | Y | Y | Critically Low |

Abbreviations: Domains, “D; Yes, "Y"; No, "N"; Partial Yes, "PY"; Not valuable, "NV"; domains with “*” are the AMSTAR-2 critical domains
